# A Single Smart Nanomaterial for Near‐Infrared‐Switchable Theranostics of Hemorrhage and Thrombosis via Photoacoustic Imaging

**DOI:** 10.1002/smsc.70366

**Published:** 2026-11-05

**Authors:** Yi Ye, Zhilu Sun, Daofu Wu, Jun’an Lai, Mingyue Yang, Zhiqiong Luo, Sha Cheng, Suwen Yang, Hong Zeng, Yinghui Li, Faguang Kuang, Baofei Sun, Wenxia Zhang, Xiaosheng Tang, Heng Luo, Guofeng Wu

**Affiliations:** ^1^ Emergency Department of the Affiliated Hospital/College of Clinical Medicine Guizhou Medical University Guiyang Guizhou P. R. China; ^2^ State Key Laboratory of Discovery and Utilization of Functional Components in Traditional Chinese Medicine Natural Products Research Center of Guizhou Province Guizhou Medical University Guiyang P. R. China; ^3^ Department of Emergency The First Affiliated Hospital Hengyang Medical School University of South China Hengyang Hunan P. R. China; ^4^ State Key Laboratory of Catalysis Dalian Institute of Chemical Physics Chinese Academy of Sciences Dalian P. R. China; ^5^ Department of Radiology Xinqiao Hospital Army Medical University Chongqing P. R. China; ^6^ College of Basic Medical Guizhou Medical University Guiyang Guizhou P. R. China; ^7^ College of Optoelectronic Engineering Chongqing University of Posts and Telecommunications Chongqing P. R. China

**Keywords:** Ag nanoparticles, Ag single atom, NiFe‐layered double hydroxides, near‐infrared switching, on‐demand hemostasis, photoacoustic imaging, theranostics

## Abstract

The clinical management of hemorrhagic patients remains a significant challenge, as current therapeutic approaches lack real‐time diagnostic capability and spatiotemporal precision. Conventional materials (e.g., hydrogels) require surgical intervention, while molecular targeting strategies are limited by slow kinetics and uncontrollable thrombotic risks. To address these limitations, we developed a smart nanomaterial based on a NiFe‐layered double hydroxide decorated with both Ag single atoms and nanoparticles (Ag_SA + NP_/NiFe‐LDH). This platform can bind plasminogen activators, achieving rapid hemostasis with approximately 30% reduced fibrinolysis in vitro and approximately 50% reduced blood loss in vivo in models of intracranial and abdominal hemorrhage. Under pulsed near‐infrared (NIR) irradiation, the dominant photoelectric effect enhances hemostatic efficacy, reducing blood loss by about 50% compared to the unexposed part; under sustained NIR exposure, strong photothermal effects inactivate clot‐enriched thrombin and alter fibrin structure, thereby promoting controlled thrombolysis. This dual functionality is consistently demonstrated in arterial models and in cryopreserved human blood samples from healthy individuals and hemorrhagic patients. Moreover, the material enables real‐time visualization of active bleeding (as low as 3 μL) via photoacoustic imaging, as validated in an intracranial hemorrhage model. Our work demonstrates a proof‐of‐concept NIR‐based strategy for externally modulated management with potential applications in dynamic vascular emergencies.

## Graphical abstract

A smart nanomaterial integrating Ag single atoms and nanoparticles on NiFe‐layered double hydroxides enables NIR‐switchable hemostasis and thrombolysis via photoelectric and photothermal effects. This platform allows real‐time photoacoustic imaging of active bleeding and exhibits rapid in vivo clearance, offering a dynamic theranostic strategy for managing life‐threatening hemorrhage.

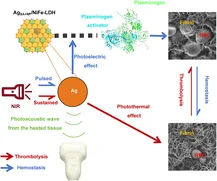

## 1 Introduction

Arterial hemorrhagic diseases, notably spontaneous intracranial hemorrhage (ICH) and traumatic hemorrhage, pose significant global health burdens, frequently leading to disability and mortality [1, 2]. Persistent bleeding is the main contributor to these outcomes, causing hematoma enlargement in spontaneous ICH [3] and substantial blood loss in trauma [4, 5].

Material‐based hemostats, such as hydrogels [6, 7], have emerged as promising alternatives. However, their application typically necessitates precise localization and often surgical exposure, rendering them impractical for emergent, noncompressible, or deep‐seated hemorrhage (e.g., intracranial or intra‐abdominal bleeding). Meanwhile, the pursuit of targeted therapeutics using ligand–receptor interactions (e.g., antibody‐directed hemostatic agents) faces a fundamental physical constraint in the context of hemorrhage. The rapid blood flow at a bleeding site severely limits the dwell time required for such biochemical recognition and binding to occur, making them ineffective for rapid hemorrhage control [8, 9, 10]. More critically, once initiated, these biochemical reactions are often self‐sustaining until the agent is exhausted, creating an uncontrollable pro‐thrombotic risk that can lead to devastating thromboembolic complications [11, 12]. Therefore, an ideal agent must combine rapid, flow‐resistant targeting with an externally tunable “off” switch to ensure safety.

While several stimuli‐responsive nanomaterials have been developed for either hemostasis or thrombolysis individually—including pH‐responsive hydrogels for clotting factor concentration, magnetically actuated microdevices for targeted drug delivery, and photothermal agents for clot ablation as mentioned above—platforms that integrate both functions in a single system remain rare (see Table S1 for a detailed comparative summary). Critically, no existing system exploits differences in irradiation temporal modality (pulsed vs. sustained near‐infrared (NIR)) to achieve bidirectional, externally switchable control over the hemostatic–thrombolytic balance.

Light‐responsive materials offer a compelling solution due to their instantaneous action and precise spatiotemporal control. However, no existing platform integrates real‐time diagnostic imaging with a bidirectional therapeutic switch to address the opposing pathologies of bleeding and clotting. Layered double hydroxides (LDHs) are a class of versatile nanomaterials with tunable catalytic properties, and recent advances have highlighted the potential of LDH‐based nanomaterials in biomedical applications, including drug delivery, imaging, and phototherapy [13, 14, 15, 16, 17, 18, 19, 20], yet their potential in hemostasis remains unexplored. We discovered that NiFe‐LDH intrinsically binds plasminogen activators (PAs), key regulators of fibrinolysis. By employing a defect‐engineering strategy to decorate NiFe‐LDH with Ag single atoms (SAs) and nanoparticles (NPs) to mitigate embolism risks [21, 22] and introduce photosensitivity [23, 24], we created Ag_SA + NP_/NiFe‐LDH. This nanoplatform uniquely harnesses the photoelectric effect of Ag SAs for instantaneous hemostatic activation and the photothermal effect of Ag NPs for controlled thrombolysis, all while serving as a photoacoustic imaging agent for real‐time hemorrhage detection. This work establishes a new paradigm of intelligent vascular theranostics, moving beyond static materials to dynamic, image‐guided, and externally regulated systems.

## 2 Results and Discussion

### 2.1 Synthesis and Characterizations of NiFe‐LDH

NiFe‐LDH was synthesized via a hydrothermal method (Figure 1a) [25]. Scanning electron microscopy (SEM) revealed its three‐dimensional (3D) structure (Figure 1b), while transmission electron microscopy (TEM) images confirmed the presence of relatively uniform nanosheets (Figure 1c,d), which ensured stable, controllable, and reproducible biological performance with enhanced safety. Figure 1e presents the high‐angle annular dark‐field scanning TEM (HAADF–STEM) image of the pristine LDH nanosheets. This characteristic structural profile serves as a fundamental reference for evaluating subsequent surface modifications. Beyond routine characterization, analysis of the dispersion stability indicated that NiFe‐LDH exhibited limited dispersibility (Figure 1f), suggesting a potential risk of embolism in vivo.

**FIGURE 1:**
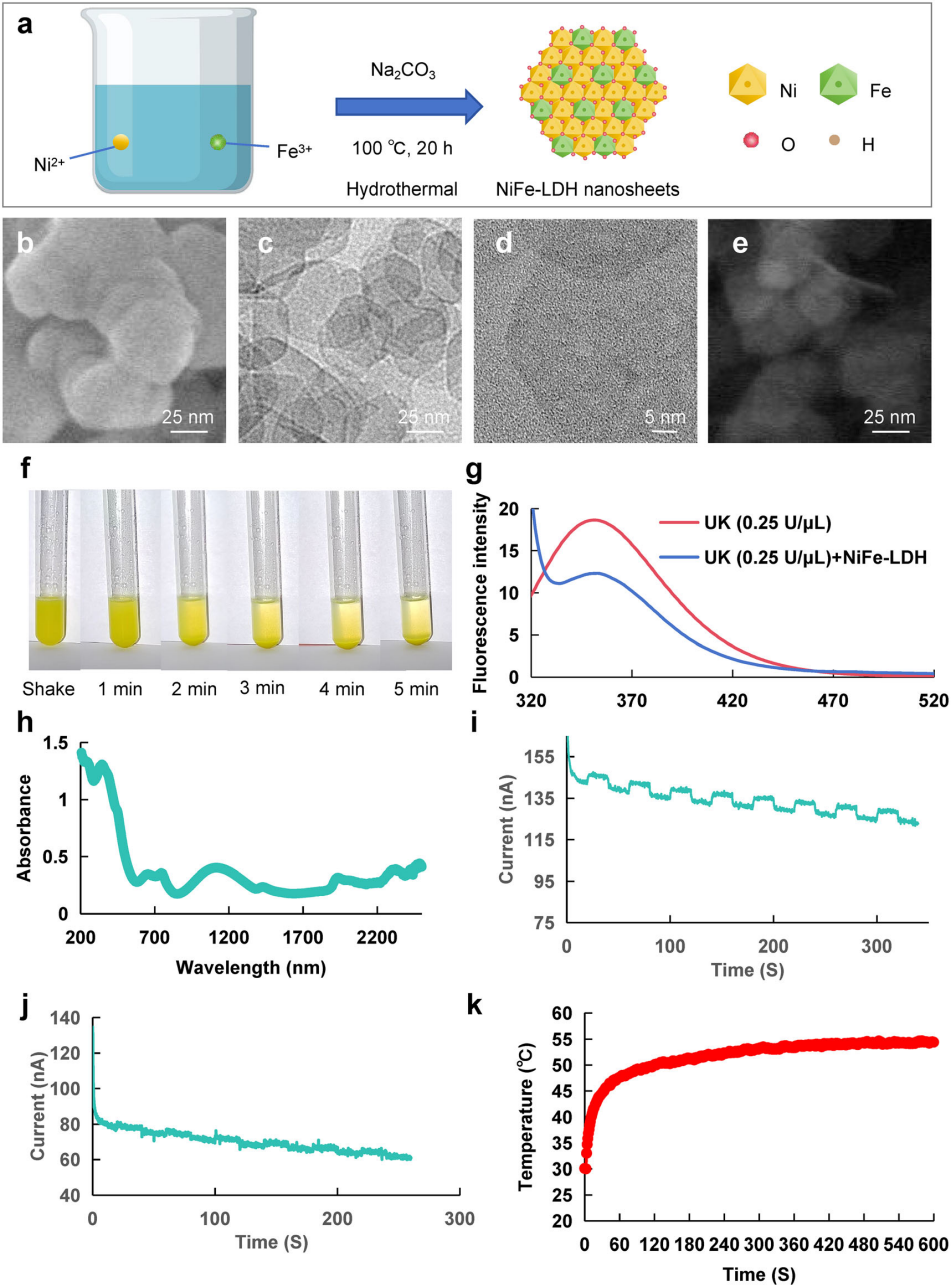
Synthesis and characterization of NiFe‐LDH. (a) Schematic of NiFe‐LDH synthesis via hydrothermal method. (b) SEM image revealing 3D morphology. (c,d) TEM images showing uniform nanosheet structure. (e) High‐angle annular dark‐field scanning transmission electron microscopy (HAADF–STEM) image confirming atomic structure. (f) Dispersion stability analysis: rapid sedimentation within 3 min indicates poor dispersibility. (g) Fluorescence quenching of UK by NiFe‐LDH (800 μg/mL): intensity decrease at *λ*_em_ ≈ 360 nm confirms binding. (h) UV–vis–NIR absorbance spectrum demonstrating broad light absorption. (i,j) Transient photocurrent responses: strong signal at 680 nm (i) and weak response at 808 nm (j). (k) Photothermal performance under 808 nm irradiation: heating curves showing temperature elevation.

Fluorescence quenching assays [26, 27, 28] were employed to investigate the interaction between NiFe‐LDH and urokinase (UK), a key PA that triggers the fibrinolytic system to dissolve thrombi and serves as a critical hemostatic target [29, 30]. UK displays characteristic tryptophan emission at approximately 360 nm. The observed decrease in fluorescence intensity upon quenching (Figure 1g) suggests potential binding between NiFe‐LDH and UK. This interaction may contribute to the therapeutic hemostatic effect, possibly through inhibition of PA activity, which could disrupt fibrinolysis at its source. Further studies are required to confirm the exact mechanism. Additionally, the photosensitive properties of NiFe‐LDH were systematically evaluated. Absorption spectroscopy revealed strong visible‐to‐NIR light absorption (Figure 1h), confirming its photosensitization potential. Photoelectrochemical characterization further demonstrated wavelength‐dependent responses: the photocurrent at the NIR region was weak (Figure 1i), suggesting that defect engineering may be required to achieve an efficient photoelectric effect under NIR irradiation. Moreover, photothermal measurements indicated limited heating capability (Δ*T* < 25 °C), necessitating further surface modifications to enhance photothermal conversion efficiency (Figure 1j,k). Figure S1 showed additional characterizations of NiFe‐LDH.

The limited dispersion stability of NiFe‐LDH poses dual clinical challenges: embolism risk from aggregation and rapid sedimentation in blood, compromising hemostatic efficacy. Surface modification is thus essential to enhance dispersion stability, potentially enabling safer intravenous delivery [21, 22]. Concurrently, we propose a synergistic photosensitization strategy by decorating NiFe‐LDH with both Ag SAs and NPs [23, 24]. While AgNPs offer NIR photothermal response [31], their photoelectric conversion is hindered by Schottky barrier‐blocked hot electron transfer. Conversely, AgSAs exhibit superior photoelectric activity but lack NIR absorption [32]. Through integrated design, AgSAs act as atomic relay stations that capture and inject hot electrons from plasmonic AgNPs into the NiFe‐LDH matrix, achieving NIR‐driven photoelectric synergy. This addresses the critical need for instantaneous thrombus initiation under arterial flow conditions.

### 2.2 Synthesis and Characterization of Ag_SA + NP_/NiFe‐LDH

Ag_SA + NP_/NiFe‐LDH was synthesized using a defect engineering method (Figure 2a) [33]. After immersion in Ag^+^ solution to allow precursor adsorption, Ag_SA + NP_/NiFe‐LDH was subjected to centrifugation and reduction under H_2_/Ar atmosphere.

**FIGURE 2:**
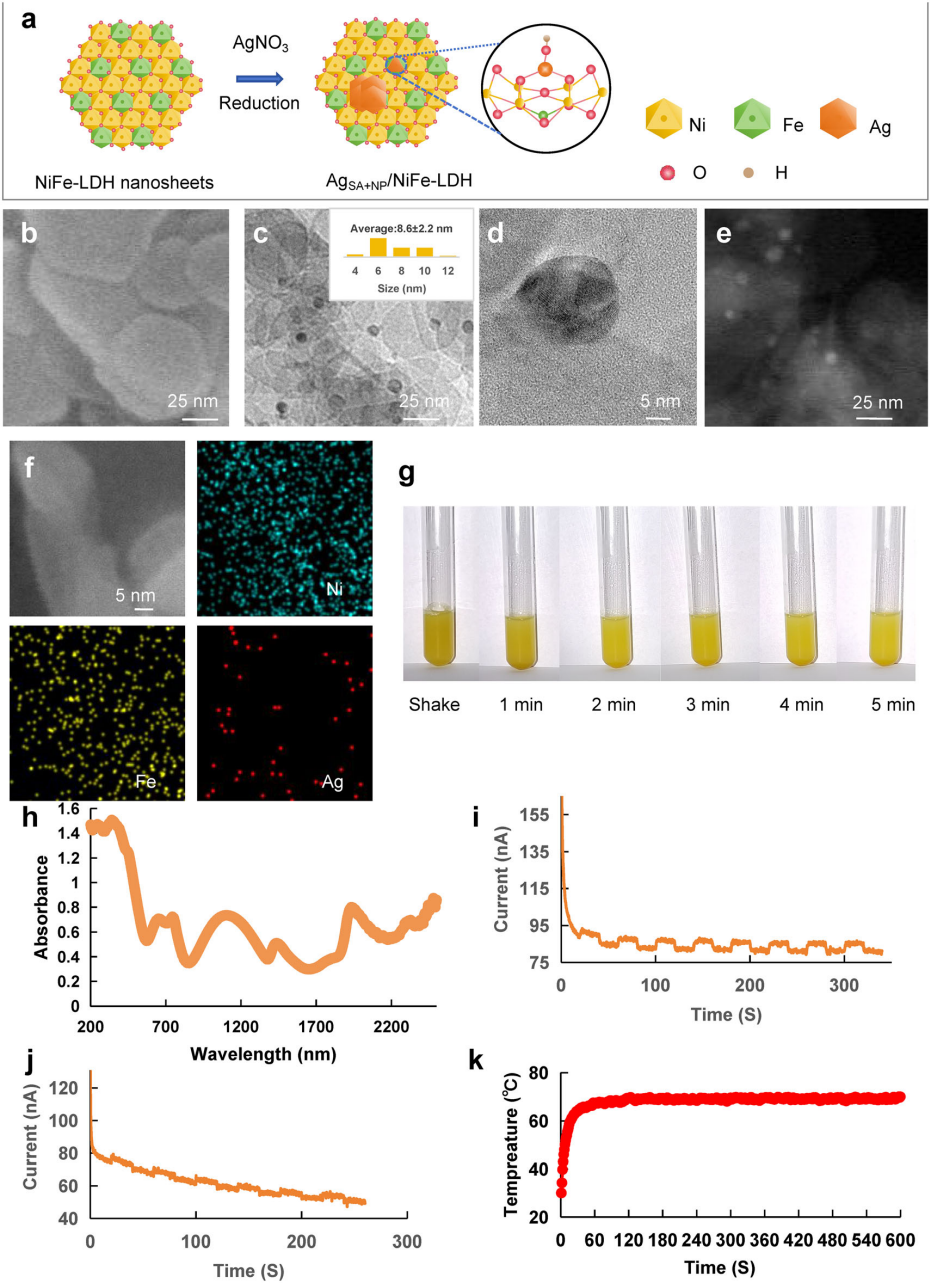
Synthesis and characterization of Ag_SA + NP_/NiFe‐LDH. (a) Synthetic scheme via defect engineering strategy. (b) SEM image showing retained 3D architecture. (c,d) TEM images confirming Ag nanoparticle (<10 nm) dispersion on nanosheets. (e) HAADF–STEM image identifying Ag NPs (highlight). (f) Corresponding elemental mapping verifying Ag SAs distribution. (g) Dispersion stability analysis: enhanced stability versus NiFe‐LDH (Figure 1f). (h) UV–Vis–NIR absorbance spectrum demonstrating broad light absorption. (i,j) Transient photocurrent responses: strong signal at 680 nm (i) and 808 nm (j). (k) Photothermal performance under 808 nm irradiation: heating curves showing temperature elevation.

SEM showed the 3D structure of Ag_SA + NP_/NiFe‐LDH (Figure 2b), consistent with NiFe‐LDH (Figure 1b). TEM imaging revealed uniformly dispersed Ag NPs with predominant sizes <10 nm anchored on nanosheets (Figure 2c,d). HAADF–STEM directly confirmed the existence of Ag NPs (Figure 2e). HAADF–STEM elemental mapping revealed atomically dispersed Ag species with diameters below 0.5 nm, consistent with Ag SAs (0.288 nm) (Figure 2f). XPS analysis (Figure S2) confirmed the metallic Ag^0^ state (Ag 3d_5_/_2_: 368.0 eV; Ag 3d_3_/_2_: 373.9 eV), indicating successful reduction under H_2_/Ar atmosphere. Critically, dispersion stability analysis demonstrated significant improvement relative to NiFe‐LDH (Figure 2g), indicating enhanced safety for intravenous administration. Photosensitivity evaluations revealed superior properties: enhanced absorption at Vis–NIR regions (Figure 2h), photoelectric response at 808 nm (Figure 2i), and photothermal effects (Δ*T* ≈ 40 °C) under 808 nm (Figure 2j,k). Figure S1 showed additional characterizations of Ag_SA + NP_/NiFe‐LDH.

To probe the synergy between Ag SAs and NPs (Figure S3), control samples (Ag_SA_/NiFe‐LDH and Ag_NP_/NiFe‐LDH) were synthesized (Paragraph S1). In UV–Vis–NIR diffuse reflectance spectroscopy (DRS), Ag_SA_/NiFe‐LDH exhibited unchanged absorption spectra compared to NiFe‐LDH, while Ag_NP_/NiFe‐LDH showed enhanced NIR absorption via localized surface plasmon resonance (LSPR) [31]. Critically, Ag_SA + NP_/NiFe‐LDH demonstrated enhanced absorption across visible and NIR regions, possibly attributed to: plasmonic NIR absorption by AgNPs, defect‐mediated visible absorption from SAs, and interfacial charge transfer creating additional electron transition pathways [32]. Moreover, SA sites also improved NP dispersion to minimize LSPR quenching. Transient photocurrent responses revealed that Ag_SA_/NiFe‐LDH generated superior photocurrent at 808 nm [34], whereas Ag_SA + NP_/NiFe‐LDH showed higher NIR (808 nm) response than Ag_NP_/NiFe‐LDH, enabling rapid activation. Photothermal conversion efficiency confirmed pronounced heating in Ag_NP_/NiFe‐LDH and Ag_SA + NP_/NiFe‐LDH compared to the pristine material and Ag_SA_/NiFe‐LDH due to LSPR dominance. Collectively, Ag_SA + NP_/NiFe‐LDH enabled on‐demand thrombus regulation by leveraging SA‐enhanced photoresponse for hemostasis initiation and NP‐dominated heating for enzymatic activity modulation.

Stability tests under physiological conditions (PBS, 37 °C, 24 h) and repeated NIR irradiation (10 cycles) showed no significant changes in UV–Vis–NIR absorption, photocurrent, or photothermal performance (Figure S4), confirming the robustness of Ag SAs and Ag NPs.

The main focus of this work is the synergy between Ag SAs and Ag NPs, but it is worth noting that the proportion of Ag SAs and Ag NPs, as well as the further particle size of Ag NPs [35], are likely to have an impact on their synergy. Therefore, further adjustment may help to optimize the material properties.

### 2.3 Hemostatic Effect of Ag_SA + NP_/NiFe‐LDH

Building on the known PA‐binding capacity of NiFe‐LDH, Ag_SA + NP_/NiFe‐LDH was confirmed to retain this functionality (Figure S5), suggesting potential hemostatic properties. Further investigation elucidated the molecular mechanism underlying its role in hemostasis: Ag_SA + NP_/NiFe‐LDH binds to tryptophan residues within PAs such as UK and streptokinase (SK) (Figure S5). This binding is consistent with inhibition of PA activity, which would suppress the conversion of plasminogen to plasmin and reduce fibrinolysis, as supported by our plasmin activity measurements (Figure S6). Figure S7 shows the effect of Ag_SA + NP_/NiFe‐LDH on four coagulation parameters—activated partial thromboplastin time (APTT), prothrombin time (PT), thrombin time (TT), and fibrinogen (FIB)—in rat plasma. Compared with the negative control (NC) group, no significant differences were observed in any of these parameters following treatment with Ag_SA + NP_/NiFe‐LDH (800 μg/mL), indicating that the material does not interfere with either the intrinsic or extrinsic coagulation pathways, nor does it affect FIB function. These results further support that the hemostatic effect of Ag_SA + NP_/NiFe‐LDH is primarily mediated through inhibition of the fibrinolytic system, rather than direct activation of the coagulation cascade.

The hemostatic efficacy of Ag_SA + NP_/NiFe‐LDH was systematically evaluated using multiple approaches: thromboelastography (TEG), fibrinolysis assays, SEM of thrombi, and in vivo hemorrhage models.

TEG assessed antifibrinolytic effects in vitro by quantifying clot strength dynamics, where the descent phase reflects fibrinolysis [36]. Under standard conditions, 800 μg/mL Ag_SA + NP_/NiFe‐LDH showed lysis at 30 min (Ly30) comparable to the NC. However, under conditions enhanced by exogenous PAs, Ag_SA + NP_/NiFe‐LDH significantly reduced Ly30, demonstrating potent inhibition of PA activity in whole blood (Figure 3a,b).

**FIGURE 3:**
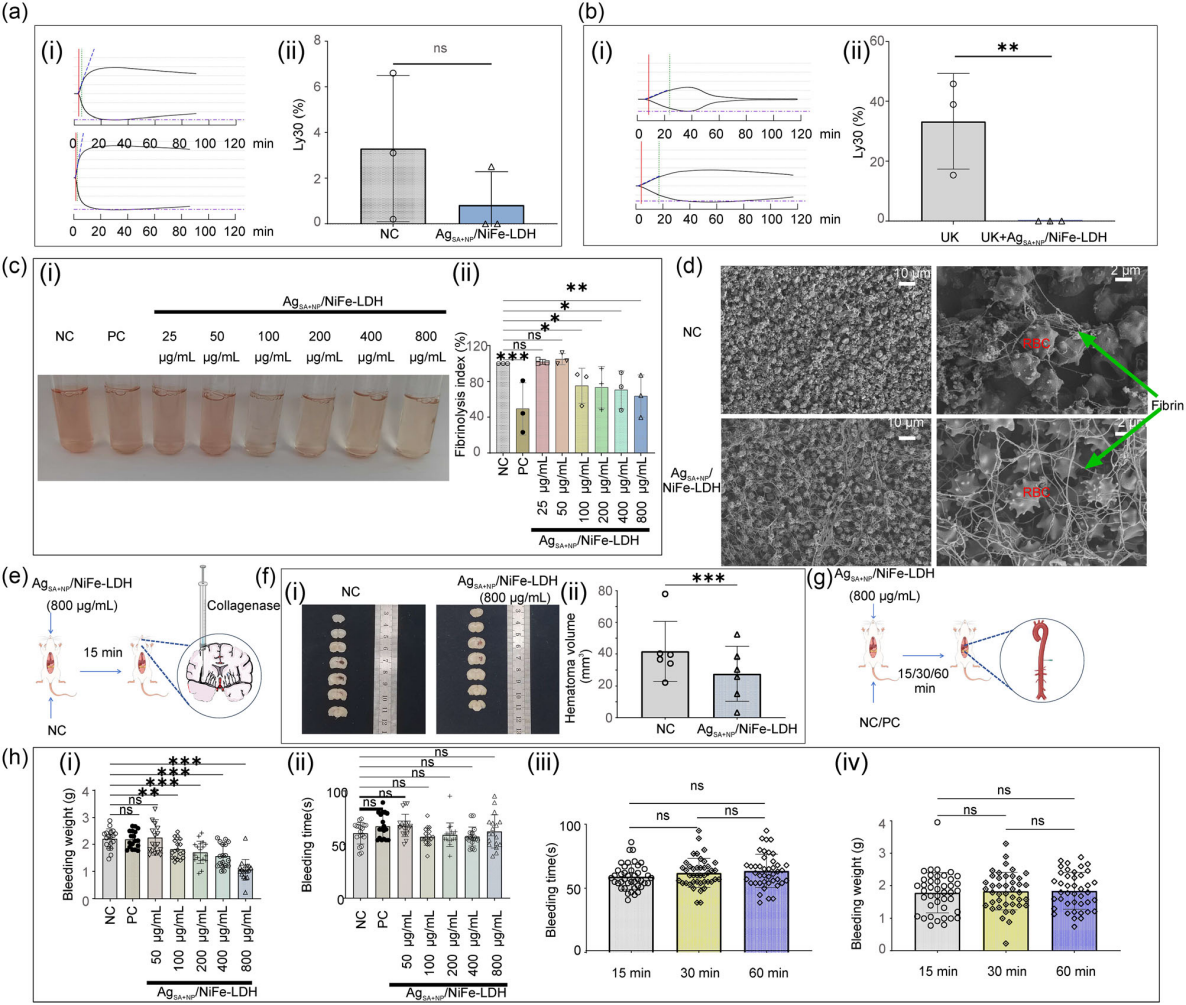
Hemostatic effect of Ag_SA + NP_/NiFe‐LDH. (a) Effect on fibrinolysis system (routine TEG). (i) Routine TEG showing that Ag_SA + NP_/NiFe‐LDH (800 μg/mL) had no significant effect. (ii) Ly30 values confirming no effect of Ag_SA + NP_/NiFe‐LDH (800 μg/mL) on fibrinolysis, data are presented as mean ± SD (*n* = 3). (b) Effect on fibrinolysis system (antifibrinolysis TEG with UK). (i) Antifibrinolysis TEG showing that Ag_SA + NP_/NiFe‐LDH (800 μg/mL) affected fibrinolysis. (ii) Ly30 values indicating a significant effect of Ag_SA + NP_/NiFe‐LDH (800 μg/mL) on fibrinolysis, data are presented as mean ± SD (*n* = 3). (c) Fibrinolysis assay**.** (i) Supernatants post fibrinolysis showed lighter color in samples with Ag_SA + NP_/NiFe‐LDH (100, 200, 400, and 800 μg/mL) compared with the NC. Representative photographs are shown; quantification is shown as the fibrinolysis index in (ii). (ii) Fibrinolysis index was lower in samples treated with Ag_SA + NP_/NiFe‐LDH (100, 200, 400, 800 μg/mL) vs. NC, data are presented as mean ± SD (*n* = 3). (d) Thrombus morphology (SEM). Compared to NC, Ag_SA + NP_/NiFe‐LDH stabilized thrombi, preserving fibrin quality and quantity while reducing red blood cell (RBC) exposure, These interpretations are based on qualitative visual assessment; formal quantitative morphometric analysis of fibrin network density and porosity remains to be performed and will be prioritized in future investigations. (e) Schematic of intracranial hemorrhage model. (f) Effect on intracranial hemorrhage. (i) Brain slices showing Ag_SA + NP_/NiFe‐LDH reduced hematoma area and accumulated layers versus NC. (ii) Ag_SA + NP_/NiFe‐LDH significantly reduced hematoma volume versus NC, data are presented as mean ± SD (*n* = 6). (g) Schematic of abdominal aortic bleeding model. (h) Effect on abdominal aortic bleeding. (i) Bleeding weight was lower in models treated with Ag_SA + NP_/NiFe‐LDH (200, 400, and 800 μg/mL) versus NC, data are presented as mean ± SD (*n* = 18). (ii) Ag_SA + NP_/NiFe‐LDH did not affect bleeding time, data are presented as mean ± SD (*n* = 18). (iii) Internal circulation times did not affect bleeding weight, data are presented as mean ± SD (*n* = 42). (iv) Internal circulation times did not affect bleeding time, data are presented as mean ± SD (*n* = 42). Wilcoxon rank sum test and Fisher's exact test were used to test group differences. ^ns^ *p* > 0.05, ^*^ *p* < 0.05, **p < 0.01, ***p < 0.001.

Fibrinolysis ability was assessed by measuring hemoglobin release from dissolving thrombi. Higher optical density and higher fibrinolysis index indicate weaker antifibrinolytic activity. Ag_SA + NP_/NiFe‐LDH (100–800 μg/mL) exhibited dose‐dependent inhibition of fibrinolysis, outperforming the NC (Figure 3c). In the fibrinolysis assay, the positive control, tranexamic acid (TXA, 100 μg/mL) reduced the fibrinolysis index of the NC. Ag_SA + NP_/NiFe‐LDH at 800 μg/mL achieved a comparable reduction, confirming potent antifibrinolytic efficacy approaching that of the clinical standard (Figure 3c). However, unlike TXA—which acts systemically as a competitive inhibitor of plasminogen binding to fibrin—our material's antifibrinolytic effect is localized to sites of existing thrombus and can be externally modulated, offering potential safety advantages.

SEM visualization of PA‐treated thrombi revealed that treatment with 800 μg/mL Ag_SA + NP_/NiFe‐LDH resulted in superior preservation of the fibrin network and reduced erythrocyte exposure compared to the NC (Figure 3d), confirming enhanced thrombus stability [36].

Following confirmation of intravenous safety (Figures S8 and S9), as well as clearance within 7 days (Figure S10), the material demonstrates a favorable safety profile. The hemostasis efficacy was validated in two severe hemorrhage models: collagenase‐induced intracranial hemorrhage model [37], in which Ag_SA + NP_/NiFe‐LDH (800 μg/mL) significantly decreased hematoma volume (Figure 3f); and aortic puncture model [38], in which Ag_SA + NP_/NiFe‐LDH (200–800 μg/mL) dose‐dependently reduced bleeding weight without affecting bleeding time (Figure 3h). The related conclusions were further testified in subgroups with different internal circulation times (Figure S11).

This progressive validation from confirming molecular binding to demonstrating efficacy in critical in vivo hemorrhage models collectively demonstrate Ag_SA + NP_/NiFe‐LDH as a novel hemostatic agent. Its unique mechanism of action operates through the inhibition of plasminogen activation. The established safety of systemic administration and its dose‐dependent efficacy highlight its therapeutic potential for managing critical hemorrhage. In addition to survival, body weight, organ weight, and histopathological evaluation (Figure S8), hematological parameters and serum biochemistry were assessed at day 14 postinjection. No significant alterations were observed in red blood cell, white blood cell, or platelet counts, hemoglobin levels, or liver and kidney function markers (Figure S9) compared with the NC group. Furthermore, macroscopic examination of the heart, lungs, liver, spleen, kidneys, and major blood vessels at necropsy revealed no visible thrombi or emboli in any animal. Together with the unchanged coagulation parameters (Figure S7), these data indicate that Ag_SA + NP_/NiFe‐LDH does not cause acute hematological toxicity, major organ dysfunction, or detectable off‐target thrombosis at the therapeutic dose within the observed period.

Regarding the potential toxicity of silver, we assessed both the total silver content and the chemical state of silver in Ag_SA + NP_/NiFe‐LDH. Inductively coupled plasma atomic emission spectrometry (ICP‐AES) analysis showed that the total silver content in the material was very low, inherently limiting the maximum possible silver exposure to biological systems (Figure S1). X‐ray photoelectron spectroscopy (XPS) analysis further confirmed that silver existed predominantly in the metallic Ag^0^ state (Ag 3d_5_/_2_: 368.0 eV; Ag 3d_3_/_2_: 373.9 eV; Figure S2), with no detectable Ag^+^ signals (Figure S2). This is a favorable safety feature, as the cytotoxicity of silver nanomaterials is primarily attributed to released Ag^+^ ions, whereas metallic Ag^0^ is relatively inert and exhibits substantially lower bioavailability and reactivity. The low total silver burden, combined with the chemically stable metallic state, suggests that Ag_SA + NP_/NiFe‐LDH poses a limited risk of silver ion–mediated toxicity at the administered dose, consistent with the absence of hematological, hepatic, or renal abnormalities observed in the 14‐day in vivo safety assessment. We fully recognize that the current safety assessment is limited to a 14‐day observation period and does not include chronic toxicity, immunotoxicity, or reproductive/developmental toxicity. These will be the focus of dedicated follow‐up studies.

### 2.4 NIR‐Switchable Targeted Hemostasis and Thrombolysis of Ag_SA + NP_/NiFe‐LDH

As mentioned, conventional targeted hemostatic materials, reliant solely on biochemical reactions, prove ineffective in high‐velocity situations due to their need for sufficient activation time. Furthermore, these reactions persist until material exhaustion, posing a significant thrombotic risk. In contrast, the photosensitive material Ag_SA + NP_/NiFe‐LDH offers dual advantages: instantaneous responsiveness and on‐demand control. Its photoelectric effects enable immediate action; current generation commenced instantly upon NIR irradiation initiation in our tests (0.25 s being the minimum measurement interval in Figure S3b,c), making it suitable for high‐velocity situations. Critically, the photoelectric effects cease immediately upon irradiation termination (current declined instantly), and while photothermal effects cause heating, this heating stops upon irradiation cessation, with the temperature gradually returning to body temperature (the specific heat capacity of Ag_SA + NP_/NiFe‐LDH is 0.355 J/g K at 42 °C (Figure S12), approximately 10% that of blood). This precise spatiotemporal control prevents uncontrolled thrombosis and downstream embolism.

Ag_SA + NP_/NiFe‐LDH was initially designed for targeted hemostasis via NIR photosensitivity, leveraging both photoelectric and photothermal effects. However, during in vitro targeted therapy under 808 nm NIR, an unexpected phenomenon emerged: pulsed NIR irradiation significantly enhanced the stability of clots formed with Ag_SA + NP_/NiFe‐LDH (reducing thrombolysis), while sustained NIR irradiation promoted their dissolution (enhancing thrombolysis). We hypothesize that pulsed irradiation favors the photoelectric effect, which may enhance the material's binding affinity for PAs via local charge accumulation at the Ag SA sites, without significant heating. Direct spectroscopic evidence for charge‐induced conformational changes in bound PAs remains to be established. Continuous irradiation leads to a dominant photothermal effect, which (1) directly inactivates heat‐sensitive thrombin within the clot and (2) denatures fibrin fibers, making the clot more susceptible to lysis. The switching behavior is governed by a shift in the dominant photoresponsive effect depending on the irradiation regimen. Pulsed NIR favors the photoelectric effect, enhancing PA binding via local charge accumulation without significant heating. In contrast, sustained NIR triggers a dominant photothermal effect, which inactivates thrombin and denatures fibrin, rendering the clot susceptible to lysis (Figure 4c).

**FIGURE 4:**
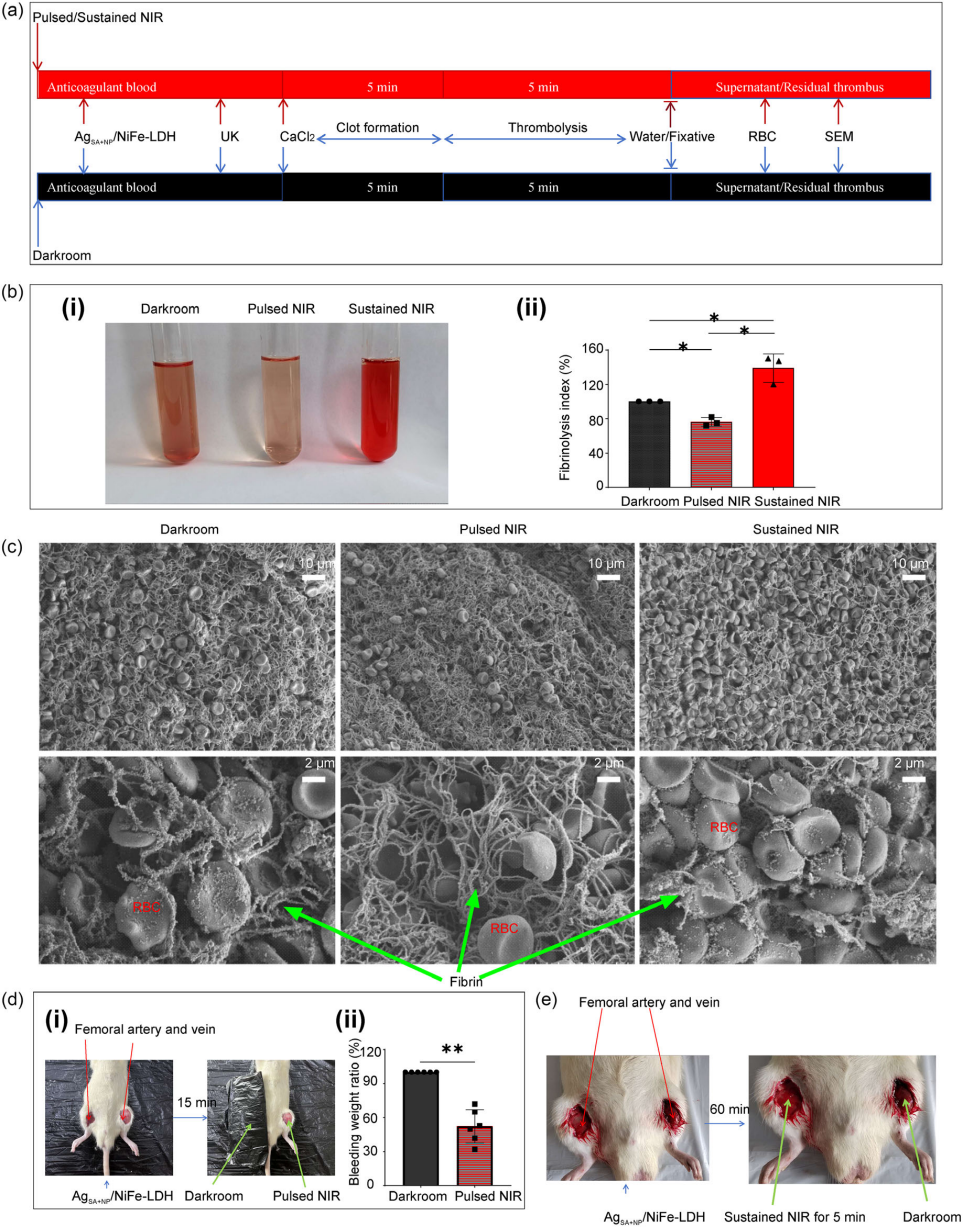
NIR‐Switchable targeted hemostasis and thrombolysis of Ag_SA + NP_/NiFe‐LDH. (a) Schematic of fibrinolysis assays and SEM analysis. (b) Effect of NIR illumination mode on antifibrinolysis (fibrinolysis assay). (i) Post fibrinolysis supernatants: Pulsed NIR group showed lighter color, while sustained NIR group showed deeper color compared to darkroom group. Representative photographs are shown; quantification is shown as fibrinolysis index in (ii). (ii) Fibrinolysis index: Lower in pulsed NIR group and higher in sustained NIR group versus darkroom group, data are presented as mean ± SD (*n* = 3). (c) Thrombus morphology (SEM). Compared to darkroom group, Ag_SA + NP_/NiFe‐LDH under pulsed NIR enhanced thrombus stability (preserving fibrin quality/quantity). Conversely, sustained NIR reduced stability. The differences in fibrin quality and quantity described here are qualitatively evident; quantitative image analysis is planned for subsequent studies to confirm these findings. (d) Hemostatic effect in bilateral femoral artery/vein bleeding models under darkroom versus pulsed NIR. (i) Schematic of model and illumination. (ii) Bleeding weight was significantly lower under pulsed NIR versus darkroom, data are presented as mean ± SD (*n* = 6). (e) Thrombolysis effect in bilateral femoral artery/vein bleeding models under sustained NIR. 60 min after hemostasis, the injuries was exposed under sustained NIR illumination for 5 min, and rebleeding was observed. Fisher's exact test were used to test group differences. ^*^ *p* < 0.05, **p < 0.01.

The targeted hemostatic function under pulsed NIR was confirmed both in vitro and in vivo using darkroom and pulsed NIR models (Figure 4a). Fibrinolysis tests demonstrated that pulsed NIR increased the material's antifibrinolytic capability by approximately 30% compared to dark conditions (Figure 4b). SEM analysis of thrombi undergoing fibrinolysis revealed superior residual fibrin network quality and quantity under pulsed NIR versus darkness, indicating enhanced thrombus stability (Figure 4c). Subsequent evaluation in a bilateral femoral artery and vein bleeding model (primarily arterial bleeding) showed pulsed NIR reduced blood loss by ~50% compared to the darkroom model (Figure 4d). Therefore, Ag_SA + NP_/NiFe‐LDH achieves targeted hemostasis through photosensitivity under pulsed NIR irradiation.

Conversely, the targeted thrombolytic function under sustained NIR irradiation was also confirmed both in vitro and in vivo using darkroom and sustained NIR models. Fibrinolysis tests showed that sustained NIR enhanced fibrinolysis by ~30% compared to dark conditions (Figure 4b). SEM assessment indicated degraded residual fibrin quality and quantity under sustained NIR versus darkness, suggesting reduced thrombus stability (Figure 4c). In the bilateral femoral bleeding model, sustained NIR triggered rebleeding (Figure 4e). Sustained NIR triggered rebleeding in all Ag_SA + NP_/NiFe‐LDH‐treated models (6/6), compared to only one rebleeding event in controls without Ag_SA + NP_/NiFe‐LDH caused by vascular dilation caused by NIR induced temperature rise (1/6; *p* < 0.05, Fisher's exact test). Therefore, Ag_SA + NP_/NiFe‐LDH achieves targeted thrombolysis through photosensitivity under sustained NIR irradiation. We propose that for deep‐seated applications, the material could be activated using minimally invasive optical fiber‐based devices, such as those already used in interstitial laser thermotherapy. For intracranial applications, a burr hole could provide access for an optical fiber during surgical or stereotactic intervention. For intra‐abdominal bleeding, laparoscopic or endoscopically delivered fibers could be employed. Moreover, our material also exhibits strong absorption in the NIR‐II region (e.g., 1064 nm, as shown in Figure 2), which offers deeper tissue penetration. We believe that by combining the material with existing interventional techniques, its clinical utility for deep‐seated pathologies can be realized.

Although the in vitro photothermal temperature reached around 70 °C, the actual in vivo temperature rise must account for the material's extremely low heat capacity and dose, local enrichment, short‐term sustained irradiation, as well as heat dissipation by blood perfusion. Based on the Pennes bioheat transfer equation, the local in vivo temperature increase is estimated to be only ~25 °C. These findings confirm the material's favorable in vivo thermal safety at the therapeutic dose. Detailed calculations are provided in Paragraph S2.

To directly interrogate the proposed photoelectric enhancement of PA binding, we monitored the fluorescence of UK in the presence of Ag_SA + NP_/NiFe‐LDH under dark and pulsed NIR conditions. UK exhibits intrinsic tryptophan fluorescence at ~360 nm, which is quenched upon binding to the material. Under pulsed NIR irradiation (808 nm, 1 s on/5 s off), the fluorescence intensity declined by an additional amount relative to the dark condition (Figure S13), indicating that NIR enhances the material–PA interaction. This enhancement is consistent with localized charge accumulation at Ag SA sites, strengthening electrostatic or coordinative binding to tryptophan residues of UK. Sustained NIR did not further increase quenching, suggesting that the photoelectric effect saturates rapidly and is not augmented by the photothermal temperature rise.

To deconvolve the contributions of photoelectric and photothermal effects, we first measured the temperature rise of the fibrinolysis system under pulsed and sustained 808 nm irradiation with the nanomaterial. Under pulsed NIR, the temperature remained nearly physiological (≈37.8 °C), whereas sustained NIR raised the bulk temperature to ≈42.3 °C (Figure S13). We then evaluated the stability of pre‐formed blood clots at these two temperatures in the absence of Ag_SA + NP_/NiFe–LDH. At 37.8 °C, the fibrinolysis index was indistinguishable from the normothermic control, ruling out any thermally driven enhancement of clot stability. In contrast, at 42.3 °C the fibrinolysis index was significantly increased, indicating that this temperature alone promotes, rather than inhibits, clot lysis. These data demonstrate that the robust antifibrinolytic effect observed under pulsed NIR cannot be attributed to bulk heating and is instead consistent with a photoelectric mechanism. Conversely, the elevated temperature achieved under sustained NIR (≈42.3 °C) may contribute to the thrombolytic outcome, which is further amplified by the localized photothermal effect of Ag_SA + NP_/NiFe–LDH at the clot–material interface. We acknowledge that direct measurement of plasmin and thrombin enzymatic activities under each irradiation mode would add further resolution. These assays are technically challenging under simultaneous NIR irradiation but are a priority for future investigation.

As summarized in Table S1, most existing nanotherapies are specialized for either hemostasis or thrombolysis, but rarely integrate both functions in a single platform. Hemostatic materials typically act by physically concentrating clotting factors or delivering procoagulant agents, yet they lack the ability to subsequently dissolve pathological thrombi [39, 40, 41]. Conversely, thrombolytic nanocarriers (e.g., tPA‐loaded systems [42]) or physical ablation agents (magnetic hyperthermia [43] and photothermal agents [44]) focus solely on clot removal, often carrying a risk of unintended bleeding. Our Ag/NiFe‐LDH platform fills this gap by offering a stimuli‐switchable dual function: it promotes hemostasis under hemorrhagic conditions via fibrinolysis inhibition (a mechanism that does not artificially activate coagulation), and upon pulsed NIR irradiation after clot formation, it generates photothermal effects to enable controlled thrombolysis. Moreover, its intrinsic photoacoustic imaging capability allows real‐time visualization, distinguishing it from most existing agents. This combination of therapeutic versatility, imaging integration, and flow‐dependent safety represents a significant advance for precision management of bleeding and thrombotic disorders.

The underlying mechanism involves a shift in the dominant effect—photoelectric versus photothermal—depending on the NIR irradiation regimen. Under pulsed NIR irradiation, the photoelectric effect predominates, primarily driving the enhanced binding capacity of Ag_SA + NP_/NiFe‐LDH to PAs. This is accompanied by only mild photothermal heating. The enhanced PA binding inhibits plasmin generation, thereby promoting clot stability. Conversely, under sustained NIR irradiation, the photothermal effect dominates. The resulting localized heating concurrently reduces the activity of key enzymes: it significantly impacts thrombin (enriched within blood clots and highly heat‐sensitive), while having a more limited effect on circulating PAs. Furthermore, the fibrin structure itself is significantly altered, making it more susceptible to degradation [45]. This combination of effects tips the balance decisively toward fibrinolysis.

This unique ability to switch between targeted hemostasis and thrombolysis via NIR modulation makes Ag_SA + NP_/NiFe‐LDH a novel material for complex clinical scenarios requiring both functions. Examples include managing myocardial infarction or cerebral infarction complicated by gastrointestinal bleeding, or treating ICH accompanied by lower limb deep vein thrombosis—situations presenting critical treatment dilemmas where conventional approaches face contradictions. Moreover, its properties enable precise thrombus management; for instance, a slightly oversized thrombus causing downstream obstruction could be selectively dissolved using sustained NIR.

A potential safety concern regarding the simultaneous potential for hemostasis and thrombolysis is mitigated by flow dependency. In high‐velocity blood flow, the instantaneous photoelectric effect primarily contributes to hemostasis. Any material incidentally warmed by irradiation (potentially initiating thrombolysis) is rapidly carried downstream by the flow before significant dissolution can occur. Conversely, significant photothermal effects driving thrombolysis only become prominent when blood flow slows sufficiently due to initial thrombus formation during hemostasis. Ag_SA + NP_/NiFe‐LDH exhibits an increasing specific heat capacity with rising temperature (Figure S12), enabling it to heat rapidly at body temperature (37 °C, specific heat capacity: 0.228 J g^−1 ^K^−1^) for efficient responsiveness, while slowing its heating rate at elevated temperatures to mitigate thermal damage. Even at the highest tested temperature (70 °C), its specific heat capacity (0.898 J g^−1 ^K^−1^) remains only one‐fourth that of blood, ensuring rapid cooling after light cessation.

Ag_SA + NP_/NiFe‐LDH functions as an NIR‐switchable material enabling targeted hemostasis under pulsed irradiation and targeted thrombolysis under sustained irradiation. This capability is highly significant for managing arterial hemorrhage (enabling rapid, precise hemostasis) and treating undesired thrombi, offering a potential solution for complex clinical situations with conflicting hemostatic and thrombolytic needs.

To address the limited tissue penetration of 808 nm light, we envision that Ag_SA + NP_/NiFe‐LDH could be activated using minimally invasive optical fiber‐based devices, such as those used in interstitial laser thermotherapy. For intracranial applications, a burr hole could provide access for an optical fiber during surgical or stereotactic intervention. For intra‐abdominal bleeding, laparoscopic or endoscopically delivered fibers could be employed.

The hemostatic mechanism of our material is based on inhibition of fibrinolysis—it stabilizes formed clots by protecting them from premature degradation, rather than actively promoting coagulation. This mechanism is inherently localized to the vascular injury site, as it requires the presence of an existing thrombus (formed via the body's natural coagulation cascade) to exert its effect. During circulation, the material encounters only intact endothelium and does not interfere with systemic fibrinolytic balance, making systemic thrombotic risk highly unlikely. Under pulsed NIR irradiation, this basal hemostatic function is spatially and temporally enhanced—the focused light beam ensures that enhanced activity occurs only at the target site (e.g., bleeding vessel), and the pulsed mode provides precise temporal control. Furthermore, a flow‐dependent safety mechanism is inherent to this system: during active hemorrhage, rapid blood flow rapidly dissipates any photothermal heating, allowing the photoelectric effect to dominate and enhance hemostasis without unintended effects. Once hemostasis is achieved and blood flow slows, the photothermal effect becomes significant, enabling subsequent controlled thrombolysis if needed. This dual‐layer safety mechanism ensures that the material's functions are deployed appropriately without off‐target risks.

### 2.5 NIR‐Switchable Targeted Hemostasis and Thrombolysis in Human Blood Samples

Clinical translation potential was evaluated using blood from healthy donors (*n* = 5) and ICH patients (*n* = 5). The NIR‐switchable functionality was robustly reproduced in human samples: under pulsed NIR, the fibrinolysis index decreased by ~30% (healthy) and ~35% (ICH) compared to dark controls, while sustained NIR enhanced fibrinolysis by ~20% (healthy) and ~25% (ICH) (Figure S14). This consistency supports the translational robustness of the NIR‐switching mechanism.

We note several important limitations in our human blood experiments that warrant cautious interpretation. First, all blood samples were cryopreserved at −80 °C, a process that inevitably impairs platelet integrity and function, thereby disrupting the physiological coagulation response. To circumvent this, thrombin was supplemented at a standardized low dose (0.5 U/mL) to achieve consistent clot formation; however, this bypasses the endogenous platelet‐dependent coagulation pathway. Second, the fibrinolysis assay is performed in a static well‐plate format, which does not replicate the shear forces, flow dynamics, and endothelial interactions present in vivo. Third, the limited blood volume obtainable from each donor under the current ethical protocol precluded more comprehensive clinical laboratory analyses (e.g., D‐dimer, plasmin–antiplasmin complexes, thrombin generation assays). These constraints are particularly acute for ICH patient samples, which are collected under urgent clinical conditions with minimal spare volume for research. Consequently, the data presented in this section should be regarded as proof‐of‐principle evidence that the NIR‐switchable mechanism is functional in human blood components, rather than as a demonstration of clinical efficacy. Future studies using fresh whole blood, flow‐chamber devices, and larger‐volume prospective sampling will be essential to bridge the gap toward clinical translation.

### 2.6 Photoacoustic Imaging for Tracking Active Hemorrhage

To further extend the functionality of Ag_SA + NP_/NiFe‐LDH from therapeutic to diagnostic, we evaluated its photoacoustic imaging [46, 47] capability in a rat ex vivo brain model simulating active hemorrhage. Upon secondary injection of the material into the cerebral tissue, strong photoacoustic signals were observed at the sites of recurrent bleeding, showing as a highlight in low background, analogous to the “spot sign” seen in clinical CTA of ICH, even with a very low amount of rebleeding at 3 μL. This allows real‐time visualization and localization of active extravasation, providing a diagnostic tool for dynamic monitoring of hemorrhage progression alongside therapeutic intervention. Given that our material is primarily designed for hemostasis with integrated imaging capability, we did not quantitatively compare its photoacoustic signal with commercial contrast agents. Since the photoacoustic signal intensity is directly proportional to the material's optical absorption coefficient, we evaluated its photostability by monitoring the UV–Vis–NIR absorption spectra and photothermal conversion performance under repeated NIR irradiation (808 nm, 10 cycles of 1 min on/off). As shown in Figure S4, the absorption spectra before and after cycling exhibited negligible change, and the photothermal temperature elevation curves remained consistent. These results indicate that the material possesses excellent resistance to photobleaching, indirectly confirming that its photoacoustic imaging signal would remain stable during repeated imaging sessions. Furthermore, the material's strong absorption in the NIR‐II region (Figure 2h), as demonstrated by our results, endows it with significant potential for photoacoustic imaging in the NIR‐II region (e.g., 1064 nm, Figure 5). This makes it a highly promising candidate for in vivo biomedical applications.

**FIGURE 5:**
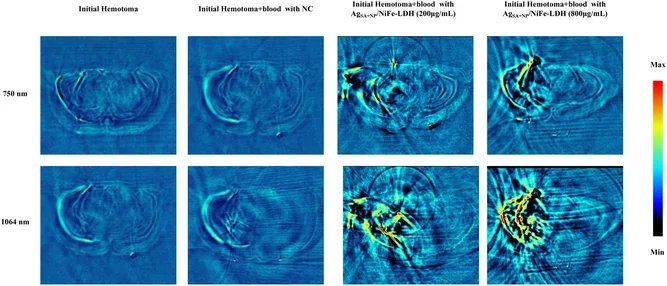
Photoacoustic imaging of intracranial hematoma and rebleeding. Photoacoustic imaging at 750 and 1064 nm showed that a hematoma formed by whole blood alone, or with the NC, could not be clearly visualized. In contrast, the administration of Ag_SA + NP_/NiFe‐LDH resulted in strong signal absorption at both wavelengths, enabling clear detection of rebleeding.

## 3 Conclusion

In this study, we developed Ag_SA + NP_/NiFe‐LDH as a single nanomaterial that integrates NIR‐switchable bidirectional therapy with photoacoustic imaging, constituting a novel theranostic platform. It enables pulsed NIR‐enhanced hemostasis and sustained NIR‐triggered thrombolysis, while simultaneously serving as a contrast agent for tracking active hemorrhage. This dual diagnostic‐therapeutic capability positions Ag_SA + NP_/NiFe‐LDH as a proof‐of‐concept platform that warrants further evaluation for the precision management of dynamic vascular emergencies, pending further validation in physiologically relevant models and rigorous safety assessment.

## 4 Materials and Methods

### 4.1 Materials and Animals

UK was sourced from Wuhan Renfu Pharmaceutical group (China). SK was obtained from Solarbio Science & Technology (China). Plasminogen fusion protein was supplied by Proteintech group, Inc. (China). Rat plasmin enzyme‐linked immunosorbent assay (ELISA) kit was obtained from Quanzhou Ruixin Biotechnology (China). Rat plasminogen ELISA kit was acquired from Wuhan Huamei Bioengineering (China). Plasmin activity assay was obtained from Abcam (the United Kingdom). Albumin/IgG removal kit was sourced from Bestbio (China). Collagenase was obtained from Sigma Aldrich (China). Thrombin solution was purchased from Mindary (China). Sprague‐Dawley rats were supplied by Chaoyu Biotechnology (China).

### 4.2 Synthesis of NiFe‐LDH

NiFe‐LDH was synthesized using a hydrothermal method. NiCl_2_·6H_2_O (15 mmol) and FeCl_3_·6H_2_O (5 mmol) were dissolved in 40 mL of deionized water under vigorous stirring until a clear solution was obtained. Subsequently, 40 mL of Na_2_CO_3_ (40 mmol) solution was added. The solution was transferred to a 120 mL Teflon‐lined stainless‐steel autoclave, sealed, and maintained at 100 °C for 20 h, and then was cooled to room temperature. The precipitate was washed three times with deionized water and ethanol, and then collected by centrifugation. After drying in an oven at 80 °C for 12 h, the desired NiFe‐LDH was obtained.

### 4.3 Synthesis of Ag_SA + NP_/NiFe‐LDH

The as‐synthesized NiFe‐LDH (100.0 mg) was first dispersed in 20 mL of deionized water containing NaOH (0.01 mmol/L) under ultrasonic vibration. Then, 4 mL of AgNO_3_ solution (1 mg/mL) was added dropwise to the LDH dispersion under stirring at room temperature. After sustained stirring for 12 h, the suspension was centrifuged and washed three times with water and ethanol. The obtained solid was dried in a vacuum oven and reduced to 5% H_2_/Ar at 100 °C for 2 h to yield the Ag_SA + NP_/NiFe‐LDH.

### 4.4 Characterization of Materials

SEM imaging was performed using a Hitachi Regulus 8100 cold‐field emission SEM (Japan) at 10.0 kV. TEM analysis was conducted on a Hitachi HT7700 instrument (Japan) operating at 100.0 kV. HAADF–STEM with energy‐dispersive X‐ray spectroscopy (EDS) mapping was carried out using a JEOL JEM‐2100F microscope (Japan) at 200.0 kV. Elemental composition was quantified by ICP‐AES on an Agilent 730 system (USA). Powder X‐ray diffraction (PXRD) patterns were acquired on a Bruker D8 ADVANCE diffractometer (Germany) with Cu Kα radiation (*λ* = 1.5418 Å). UV–Vis–NIR DRS measurements utilized a Shimadzu UV‐3600i Plus spectrophotometer (Japan). Transient photocurrent responses were recorded on a CHI660E electrochemical workstation (Chenhua, China). Photothermal conversion efficiency was evaluated using an FLIR T530 thermal imaging system under the 808 nm light source with the power of 0.1 W/cm^2^ (Pumeng, China). The specific heat capacity was measured using a Mettler DSC1 (Switzerland).

To test the dispersity, the relative materials were added into sodium acetate solution (0.1 mmol/L) at a concentration of 2000 μg/mL, then the test tube was shaken and left undisturbed for 5 min.

### 4.5 Mechanism of Ag_SA + NP_/NiFe‐LDH Interaction with PA

The mechanistic action of Ag_SA + NP_/NiFe‐LDH on PAs was progressively evaluated from purified protein systems to whole blood models. Prior to analysis, Ag_SA + NP_/NiFe‐LDH suspensions were prepared in sodium acetate buffer (0.1 mmol/L) at concentrations spanning 25–1600 μg/mL. Sprague‐Dawley rats underwent abdominal aortic blood collection under anesthesia followed by euthanasia, with citrated whole blood prepared via 1:9 sodium citrate addition. Critically, all blood aliquots within a given experimental series were derived from a single collection tube to ensure biological consistency across reagent treatments. Sodium acetate buffer served as NC and TXA‐sodium acetate solution (120 U/mL) as PC, with all animal protocols approved by our Institutional Animal Ethics Committee.

At the purified protein level, fluorescence spectroscopy (F‐320 spectrofluorometer, Tianjin Gangdong) and UV‐Vis spectroscopy (Cary 60, Agilent) characterized interactions: emission spectra of UK and SK were recorded at 285 nm excitation (10.0 kV, 37 °C) across concentration gradients [48], with quenching mechanisms analyzed via Stern–Volmer kinetics and lifetime decay [49]. Additional spectral analyses at 280 nm excitation assessed complexes of Ag_SA + NP_/NiFe‐LDH with plasminogen and UK.

Transitioning to whole blood systems, given the material's PA targeting, plasminogen (substrate) and plasmin (product) levels were quantified through parallel methodologies. For ELISA and chromogenic assays, test systems containing 1 mL citrated blood + 150 μL Ag_SA + NP_/NiFe‐LDH (800 μg/mL) or NC + 45 μL UK (1 U/μL) were incubated at 37 °C, clotted with 50 μL CaCl_2_, incubated in water‐bath for 5 min), centrifuged at 5000 × *g*, 15 min), and the resulting supernatants were analyzed per kit protocols; the ELISA specifically established UK dose‐plasmin response relationships. Western blot analysis employed five experimental groups with the same volume of all reaction systems: (1) 1 mL citrated blood, (2) blood + UK (45 μL, 1 U/μL), (3) blood + UK + Ag_SA + NP_/NiFe‐LDH (150 μL, 800 μg/mL), (4) blood + UK + NC (150 μL), and (5) blood + Ag_SA + NP_/NiFe‐LDH. Following identical clotting driven by CaCl_2_/centrifugation processing and albumin/IgG depletion, plasminogen was quantified against transferrin reference via ImageJ 2.3.0 after electrophoretic separation.

### 4.6 Method for Coagulation Assays

To evaluate whether Ag_SA + NP_/NiFe‐LDH itself affects the coagulation pathway, four coagulation parameters—APTT, PT, TT, and FIB—were measured. Briefly, rats were anesthetized, and blood samples were collected via cardiac puncture and anticoagulated with sodium citrate. The anticoagulated whole blood was then centrifuged to obtain platelet‐poor plasma. Subsequently, Ag_SA + NP_/NiFe‐LDH (800 μg/mL) or NC was added to the plasma at a ratio of 10% (v/v). The mixtures were incubated at 37 °C with gentle shaking for 5 min, followed by the measurement of coagulation parameters using an automated coagulation analyzer.

### 4.7 Hemostatic Effect of Ag_SA + NP_/NiFe‐LDH

The hemostatic efficacy of Ag_SA + NP_/NiFe‐LDH was evaluated through TEG assessing antifibrinolytic activity, quantitative fibrinolysis assays with SEM thrombus visualization in vitro, and arterial hemorrhage models in vivo. TEG analysis employed a Haema T4 analyzer (Shenzhen Medcaptain Biomedical Technology) under standard and PA‐enhanced conditions. For routine TEG, 150 μL of 800 μg/mL Ag_SA + NP_/NiFe‐LDH/NC was added to 1 mL citrated whole blood; after 37 °C equilibration, kaolin activator and 20 μL CaCl_2_ were sequentially introduced before transferring 340 μL blood–kaolin mixture to the reaction cup. Antifibrinolysis TEG followed identical protocols with prior addition of 45 μL UK (1 U/μL) to blood.

Fibrinolysis quantification involved incubating 0.2 mL blood with 20 μL test material (Ag_SA + NP_/NiFe‐LDH/NC/PC) and 25 μL UK (1 U/μL) for 15 min at 37 °C, followed by clot induction with 20 μL CaCl_2_ (5 min, 37 °C). After 5 min fibrinolysis, reactions were terminated with 15 mL ultrapure water. Samples were oscillated (50 rpm, 37 °C, 5 min), centrifuged (1000 × *g*, 5 min), and the supernatant absorbance was measured at 545 nm (Synergy 2 microplate reader, Gene Company). Fibrinolysis index (%) was calculated as (sample OD/NC OD) × 100%, with triplicate determinations.

SEM sample preparation combined 1 mL citrated blood with 150 μL Ag_SA + NP_/NiFe‐LDH (800 μg/mL)/NC and 45 μL UK (1 U/μL), which was incubated 15 min at 37 °C. Clots formed after adding 50 μL CaCl_2_ were fixed immediately, incubated 5 min at 37 °C, and imaged using cold‐field emission SEM (Hitachi Regulus 8100, 3.0 kV).

### 4.8 Arterial Hemorrhage Validation Utilized Two Models

**Aortic puncture**: Anesthetized rats underwent abdominal aortic exposure and puncture 1 cm distal to the left renal vein. Subjects with bleeding volume >20% of total blood volume were excluded; matched cohorts received intravenous injections of tested materials (1.43 μL/g body weight) via the inferior vena cava. After 15/30/60 min circulation, repuncture at the abdominal aortic origin measured bleeding weight/time prior to euthanasia.

**ICH**: Rats received tail vein injections of 800 μg/mL Ag_SA + NP_/NiFe‐LDH/NC (1.43 μL/g). After 15 min, collagenase (0.12 U) was stereotactically injected into the striatum (Ruiwode 68025 stereotaxic device) to induce ICH. Following 24 h, hematoma volumes were quantified on brain cryosections using ImageJ 2.3.0.

### 4.9 Safety of Intravenous Ag_SA + NP_/NiFe‐LDH Administration

To evaluate systemic safety, rats received tail vein injections of 1600 μg/mL Ag_SA + NP_/NiFe‐LDH solution or NC at 1.43 μL/g body weight and were maintained under standard housing conditions for 2 weeks prior to euthanasia. Body weight gain, absolute organ weights (heart, liver, spleen, lungs, kidneys), and histopathological status were comparatively assessed; organ samples underwent hematoxylin and eosin (H&E) staining per standardized protocols (Servicebio Biotechnology, China) followed by whole‐slide digital scanning using a 3DHISTECH Pannoramic MIDI system (Hungary) for comprehensive tissue morphology analysis.

For hematology and biochemistry analysis, whole blood was collected via cardiac puncture at day 14 postinjection. Complete blood count was performed using an automated hematology analyzer (Sysmex XN‐1000V). For serum biochemistry, blood was centrifuged (3000 × *g*, 10 min), and the supernatant was analyzed using a Roche Cobas c702 automated chemistry analyzer.

### 4.10 Biodistribution and Clearance Study

To investigate the in vivo fate of Ag_SA + NP_/NiFe‐LDH, Sprague‐Dawley rats received a single intravenous injection of the material (1600 μg/mL, 1.43 μL/g body weight) via the tail vein. At predetermined time points (1 h and 7 days postinjection), rats were anesthetized and euthanized. Major organs (heart, liver, spleen, lungs, and kidneys) and whole blood were collected. Tissue samples were weighed and digested in aqua regia at 80 °C until complete dissolution. The nickel (Ni) content in each sample was quantified by inductively coupled plasma mass spectrometry (ICP‐MS) as a tracer for the material, given the low Ag content and endogenous iron background. The relative Ni content in the whole body at each time point was calculated as a percentage of the total injected dose.

### 4.11 Targeted Hemostasis and Thrombolysis Under NIR Modulation

The pulsed NIR hemostasis evaluation comprised in vitro fibrinolysis assays and bilateral femoral vessel hemorrhage models. Fibrinolysis tests adapted the established protocol: test systems contained 0.2 mL citrated blood + 20 μL Ag_SA + NP_/NiFe‐LDH (800 μg/mL) + 25 μL UK (1 U/μL) + 20 μL CaCl_2_. Dark controls underwent complete light exclusion via blackout enclosures until ultrapure water addition, while pulsed NIR groups received 808 nm irradiation (12 W source, 1 cm^2^ initial spot, 5 cm distance; 1 s on/5 s off duty cycle). SEM samples prepared with 1 mL blood + 150 μL Ag_SA + NP_/NiFe‐LDH (800 μg/mL) + 45 μL UK + 50 μL CaCl_2_ followed identical photomodulation protocols until fixative addition. For in vivo validation, rats received tail vein injections of 800 μg/mL Ag_SA + NP_/NiFe‐LDH (1.43 μL/g), followed after 15 min circulation by bilateral femoral vessel transection. Immediate light exclusion (non‐contact blackout enclosure) defined the dark group, while pulsed NIR (identical parameters) illuminated the contralateral wound; bleeding mass after 5 min quantified hemostatic efficacy.

Conversely, sustained NIR thrombolysis assessment mirrored this experimental framework with irradiation modification. Fibrinolysis test systems (0.2 mL blood + 20 μL material + 25 μL UK + 20 μL CaCl_2_) and SEM preparations (1 mL blood + 150 μL Ag_SA + NP_/NiFe‐LDH + 45 μL UK + 50 μL CaCl_2_) employed sustained 808 nm irradiation (12 W, 1 cm^2^ spot, 5 cm distance) versus dark controls. The bilateral femoral vessel transection models replicated injection and transection protocols; after spontaneous hemostasis, sustained NIR exposure to one wound versus light‐shielded contralateral control enabled continuous rebleeding monitoring for 5 min to quantify thrombolytic activity.

NIR‐enhanced PA binding (fluorescence quenching). UK (0.5 mg/mL) was mixed with Ag_SA + NP_/NiFe‐LDH (800 µg/mL) and incubated at 37 °C for 10 min. Fluorescence emission spectra (*λ*_ex_ = 285 nm, *λ*_em_ = 300–500 nm) were recorded using an F‐320 spectrofluorometer under dark, pulsed NIR (808 nm, 1 s on/5 s off), and sustained NIR conditions. The NIR fiber was positioned 5 cm from the cuvette, and spectra were acquired immediately after 2 min of irradiation to avoid photobleaching.

Clot lysis under external heating. Clots were formed from 200 μL citrated rat blood + 20 μL CaCl_2_ (0.2 M) + UK without any nanomaterial. Clots were incubated exposed to different tempreatures for 10 min. Supernatant absorbance at 545 nm was measured to calculate fibrinolysis index.

### 4.12 NIR‐Switchable Hemostasis and Thrombolysis in Human Blood Samples

Ethylenediaminetetraacetic acid‐anticoagulated blood samples from five healthy volunteers and five intracranial hemorrhage (ICH) patients were cryopreserved at −80 °C,with all protocols approved by our Institutional Review Board (ethics oversight for human subjects). Fibrinolysis assays constituted the primary analytical approach, with critical adherence to single‐donor aliquoting per experimental series to ensure biological consistency. Prior to evaluating NIR‐switchability, baseline antifibrinolytic effects were assessed using adapted protocols: test systems contained 0.1 mL thawed whole blood + 20 μL Ag_SA + NP_/NiFe‐LDH/NC (800 μg/mL) + 20 μL UK (1 U/μL) + 50 μL CaCl_2_ (0.2 mol/L). Given that cryopreservation induces platelet lysis and functional inactivation, compromising natural thrombosis, we introduced thrombin supplementation (final 0.5 U/mL) to bypass platelet‐dependent coagulation; this modification enables direct interrogation of NIR‐modulated material effects on fibrinolytic pathways. Subsequent NIR‐switchability testing maintained identical reagent compositions, with darkroom controls under light‐proof enclosures versus NIR irradiation (808 nm, 12 W, 1 cm^2^ spot, 5 cm distance; pulsed: 1 s on/5 s off for hemostasis, sustained for thrombolysis) applied until reaction termination.

### 4.13 Photoacoustic Imaging for Tracking Active Hemorrhage

The photoacoustic imaging was entrusted to Zhuoxinya Technology Co., Ltd (Suzhou, China). Briefly, rat brain tissue was harvested and an initial hematoma was created by slow intracerebral injection of 10 μL whole blood. Photoacoustic images were then acquired under irradiation at 750 and 1064 nm. Subsequently, 3 μL of blood containing either Ag_SA + NP_/NiFe‐LDH (800 or 200 μg/mL)/NC was slowly injected into the preformed hematoma site. Photoacoustic imaging was repeated at the same wavelengths to assess the contrast enhancement.

### 4.14 Statistical Analysis

All experiments were performed with at least three independent biological replicates, unless otherwise stated. Sample sizes (*n*) are indicated in individual figure legends. Data are presented as mean ± standard deviation (SD). For comparisons between two groups, a two‐tailed Student's t‐test was used with Welch's correction for unequal variances where appropriate. For multiple group comparisons, one‐way or two‐way ANOVA was used followed by Tukey's or Dunnett's post‐hoc test as specified. Fisher's exact test was used for categorical data (e.g., rebleeding events). Given the small sample sizes and the associated instability of standardized effect size estimates, formal effect sizes (Cohen's d, partial *η* ^2^) and their 95% confidence intervals were not calculated for the present analyses. Statistical significance was set at *p* < 0.05. All analyses were performed using GraphPad Prism 9.0.

## Author Contributions

**Guofeng Wu**, **Heng Luo**, **Xiaosheng Tang**, and **Baofei Sun** conceived the idea. **Daofu Wu** and **Yi Ye** completed the synthesis and characterization of materials. **Yi Ye** and **Zhilu Sun** testified the mechanism. **Yi Ye**, **Jun’an Lai**, **Mingyue Yang**, **Zhiqiong Luo**, **Yinghui Li**, **Suwen Yang**, **Sha Cheng**, **Hong Zeng**, **Faguang Kuang**, and **Wenxia Zhang** completed efficacy verification. **Yi Ye** wrote the manuscript. **Guofeng Wu**, **Heng Luo**, **Xiaosheng Tang**, and **Jun’an Lai** reviewed the manuscript.

## Funding

This study was supported by the Natural Science Foundation of China (82460838, 82260244, 62375032, 22408362), the Cultivation Program of NSFC (gyfynsfc [2022]‐1), the Natural Science Foundation of Chongqing (CSTB2023TIAD‐KPX0017), the Guizhou Provincial Committee Organization Department (QKHPTRC‐gCC[2022]034‐1), the Open Fund of the State Key Laboratory of High Field Laser Physics (Shanghai Institute of Optics and Fine Mechanics), China National Postdoctoral Program for Innovative Talents (BX20230355), China Postdoctoral Science Foundation (2024M753165), the Leading Discipline Program of The Affiliated Hospital of Guizhou Medical University (gyfyxkyc‐2023‐05), Key lab of acute brain injury and function repair in Guizhou Medical University ([2024] fy0071), and the Natural Science Foundation of Hunan Province (2026JJ82200).

## Ethical Statement

The experiments about animals were approved by the Animal Ethics Committee of Guizhou Medical University (No. 2201617), and the experiments about blood samples from volunteers and patients were approved by the Ethics Committee of Guizhou Medical University Affiliated Hospital (No. 2022717).

## Conflicts of Interest

The authors declare no conflicts of interest.

## Supporting information

Supplementary Material

## Data Availability

Research data are not shared.
