# Supplementary material for: A Single Smart Nanomaterial for Near‐Infrared‐Switchable Theranostics of Hemorrhage and Thrombosis via Photoacoustic Imaging
Source: Small Sci. 2026 Nov 5;6(11):e70366. doi: 10.1002/smsc.70366 (PMC13740492; doi:10.1002/smsc.70366)
Supplement: Supplementary file 1 [file SMSC-6-e70366-s001.pdf]

**Supplementary Table S1. Comparison of Representative Nanotherapies for Hemorrhage and Thrombosis.**

| Nanoplatform | Function | Mechanism | Stimuli-responsive | Imaging capability | Key Advantages / Limitations |
|---|---|---|---|---|---|
| This work: $Ag_{SA+NP}$/NiFe-LDH | Dual (hemostasis & thrombolysis) | Hemostasis via fibrinolysis inhibition; Thrombolysis via NIR photothermal effect | NIR light (pulsed and sustained) | Photoacoustic imaging | ✓ Stimuli-switchable dual functions<br>✓ Flow-dependent safety (hemorrhage vs. stasis)<br>✓ Integrated imaging<br>○ Mechanism avoids active coagulation activation |
| Zeolite/clay-based hemostats (e.g., QuikClot) | Hemostasis only | Physical absorption of water, concentration of clotting factors | None | None | ✓ Rapid hemostasis in trauma<br>✗ No thrombolytic function<br>✗ Exothermic reaction may cause tissue damage |
| Fibrinogen/thrombin-loaded hydrogels | Hemostasis only | Provision of coagulation factors to wound site | None (or shear) | None | ✓ Mimics natural clot formation<br>✗ No thrombolytic function<br>✗ Potential immunogenicity |
| tPA-loaded nanocarriers (liposomes, polymers) | Thrombolysis only | Enzymatic fibrinolysis (plasminogen activation) | Shear, pH, enzyme, or magnetic field | MRI/optical possible | ✓ Targeted thrombolysis<br>✗ No hemostatic function<br>✗ Systemic bleeding risk |
| Magnetic nanoparticles (e.g., $Fe_3O_4$) | Thrombolysis only | Magnetic hyperthermia or mechanical disruption | Alternating magnetic field | MRI | ✓ Deep tissue penetration (magnetic field)<br>✗ No hemostatic function<br>✗ Requires specialized |

| Nanoplatform | Function | Mechanism | Stimuli-responsive | Imaging capability | Key Advantages / Limitations |
|---|---|---|---|---|---|
| | | | | | equipment |
| Photothermal agents (e.g., gold nanorods) | Thrombolysis only | Photothermal ablation of thrombus | NIR light | Photoacoustic/thermal imaging | ✓ Remote activation<br>✗ No hemostatic function<br>✗ Potential thermal damage to surrounding tissue |

**Paragraph S1.** Synthesis of $Ag_{SA}$/NiFe-LDH and $Ag_{NP}$/NiFe-LDH.

**Synthesis of $Ag_{SA}$/NiFe-LDH**

The as-synthesized NiFe-LDH (100.0 mg) was first dispersed in 20 mL of deionized water containing NaOH (0.01 mmol/L) under ultrasonication. Then, 0.25 mL of $AgNO_3$ solution (1 mg/mL) was added dropwise to the LDH dispersion under stirring at room temperature. After stirring for 12 h, the suspension was centrifuged and washed three times with water and ethanol. The obtained solid was dried under vacuum and subsequently reduced under 5% H2 /Ar at 100 °C for 2 h to afford the AgSA/NiFe-LDH.

**Synthesis of $Ag_{NP}$/NiFe-LDH**

The as-synthesized NiFe-LDH (100.0 mg) was first dispersed in 20 mL of deionized water under ultrasonication. Then, 5 mL of a suspension containing 10 nm Ag NPs (0.1 mg/mL, pH=8) was added dropwise to the LDH dispersion under stirring at room temperature. After stirring for 12 h, the suspension was centrifuged and washed three times with water and ethanol. The obtained solid was dried under vacuum.

**Paragraph S2.** ***In Vivo*** **Temperature Rise Calculation (Pennes Bioheat Equation)**

| Parameter | Symbol | Value |
|---|---|---|
| Administered concentration | Cm | 800 μg/mL |
| Injected volume | Vinj | 0.3 mL |
| Total mass of materials | m | 480 μg |
| Specific heat capacity of material | cm | $0.36\ J\cdot g^{-1}\cdot K^{-1}$ |
| Specific heat capacity of tissue | ct | $3.6\ J\cdot g^{-1}\cdot K^{-1}$ |
| Density of tumor tissue | ρt | $1.05\ g\cdot cm^{-3}$ |
| **Laser Parameters** | | |
| Laser power density | Q | $1.0\ W\cdot cm^{-2}$ |
| Irradiation area | A | $1\ cm^2$ |
| **Physiological Parameters** | | |
| Blood perfusion rate | ωb | $0.02\ s^{-1}$ |
| Specific heat capacity of blood | cb | $3.6\ J\cdot g^{-1}\cdot K^{-1}$ |
| Density of blood | ρb | $1.06\ g\cdot cm^{-3}$ |
| Arterial blood temperature | Ta | 37℃ |
| Initial tissue temperature | T0 | 37℃ |

We obtained the corresponding parameters of the formula based on the actual situation and theoretical values, and the results showed that the $\Delta T$ =25.2 ℃. However, in practical situations, the material will not fully accumulate to the bleeding point, and blood flow will take away some heat. Moreover, the continuous irradiation time we use does not exceed five minutes, so it is basically impossible to reach 60 ℃.

**Fig. S1. The extended characterization of NiFe-LDH and $Ag_{SA+NP}$/NiFe-LDH.**

a

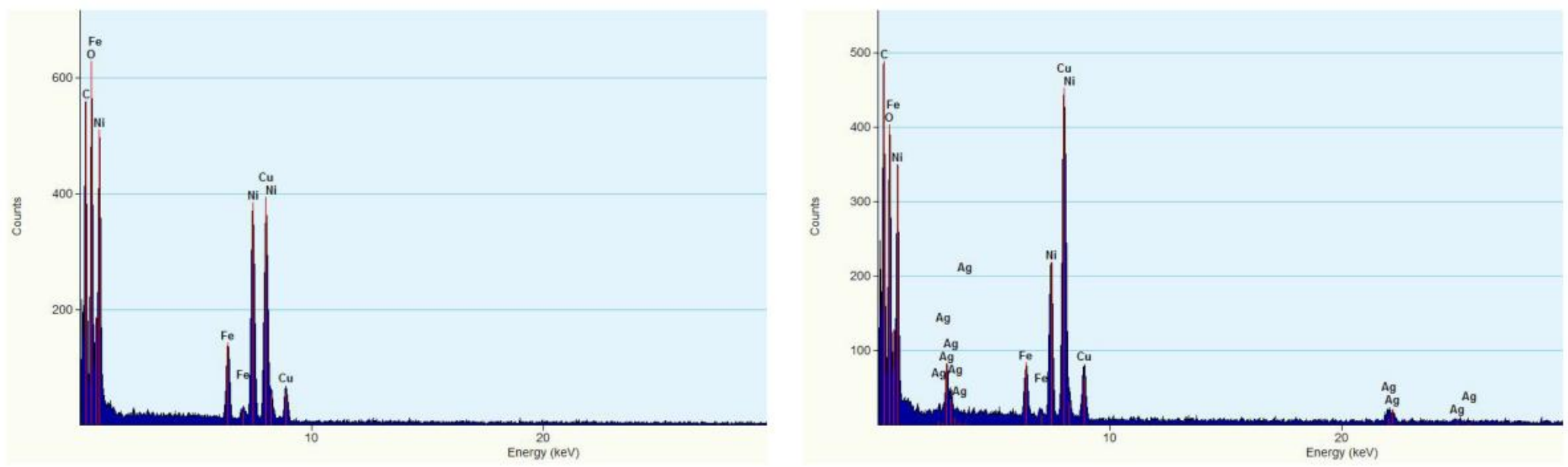


b

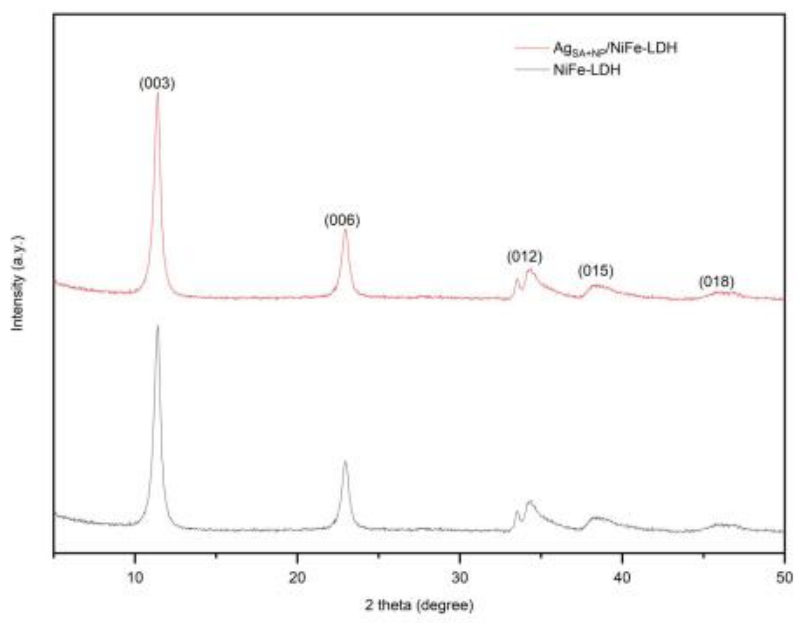


c

| Samples | Elements | Mass fraction % |
|---|---|---|
| NiFe-LDH | Fe | 10.846 |
| NiFe-LDH | Ni | 34.045 |
| $Ag_{SA+NP}$/LDH | Ag | 0.260 |
| $Ag_{SA+NP}$/LDH | Fe | 10.907 |
| $Ag_{SA+NP}$/LDH | Ni | 36.051 |

**a,** EDS. **(i)** NiFe-LDH; **(ii)** $Ag_{SA+NP}$/NiFe-LDH, confirming successful Ag loading. **b,** XRD patterns. $Ag_{SA+NP}$/NiFe-LDH maintains the characteristic peaks of NiFe-LDH with no impurity phases. No metallic Ag peaks were detected, consistent with the low Ag content. **c,** ICP-AES. Quantitatively verified the low Ag content in $Ag_{SA+NP}$/NiFe-LDH.

**Fig. S2. XPS spectrum of Ag 3d in $Ag_{SA+NP}$/NiFe-LDH.**

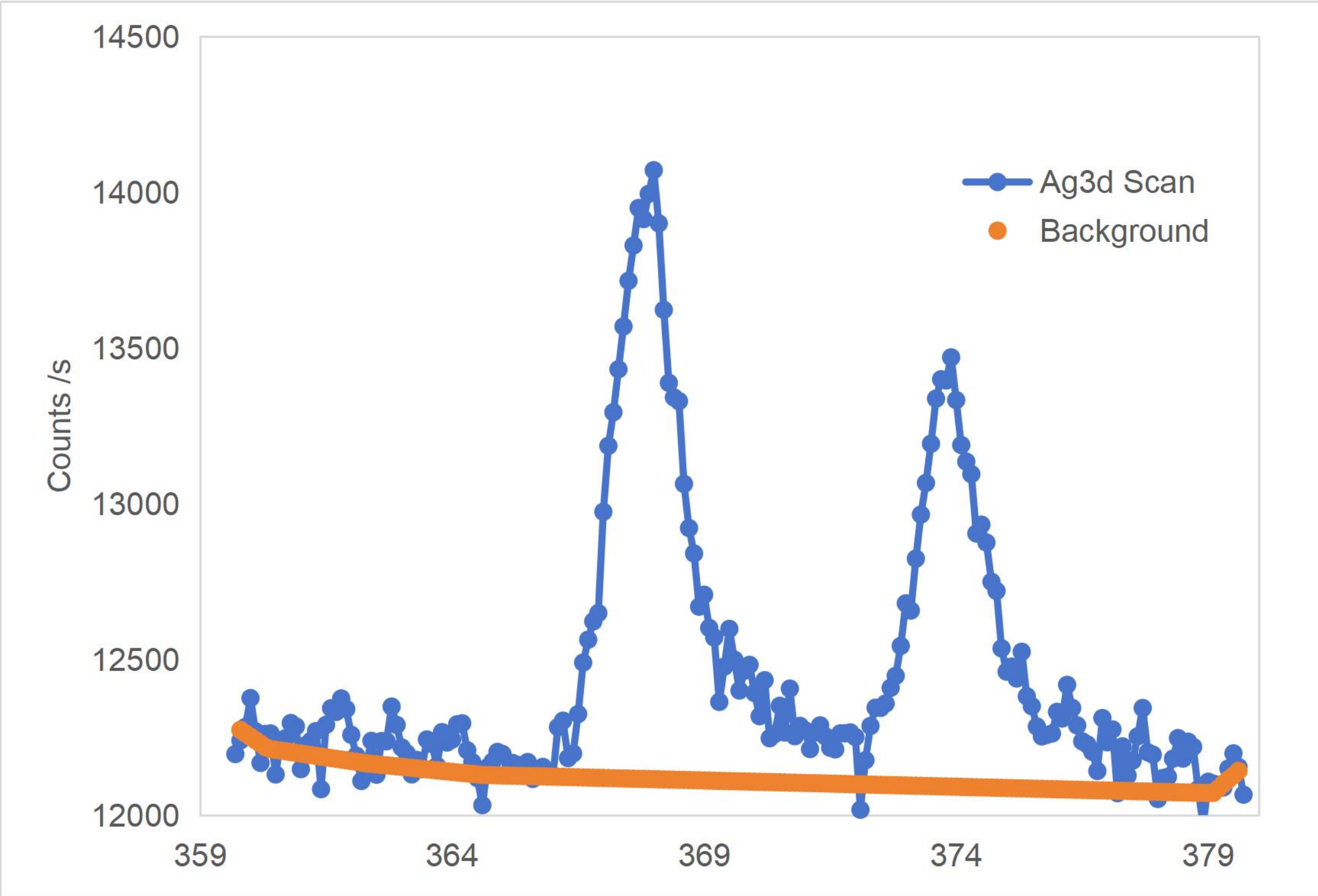


The Ag 3d spectrum exhibits two peaks at approximately 368.0 eV (Ag $3d_{5/2}$) and 373.9 eV (Ag $3d_{3/2}$), with a spin-orbit splitting of 5.9 eV. This splitting is consistent with the standard value of 6.0 eV for metallic $Ag^0$, confirming that both Ag nanoparticles and atomically dispersed Ag species are in the reduced metallic state after $H_2$/Ar reduction.

**Fig. S3. Photosensitivity property of NiFe-LDH, $Ag_{NP}$/NiFe-LDH, $Ag_{SA}$/NiFe-LDH, and $Ag_{SA+NP}$/NiFe-LDH.**

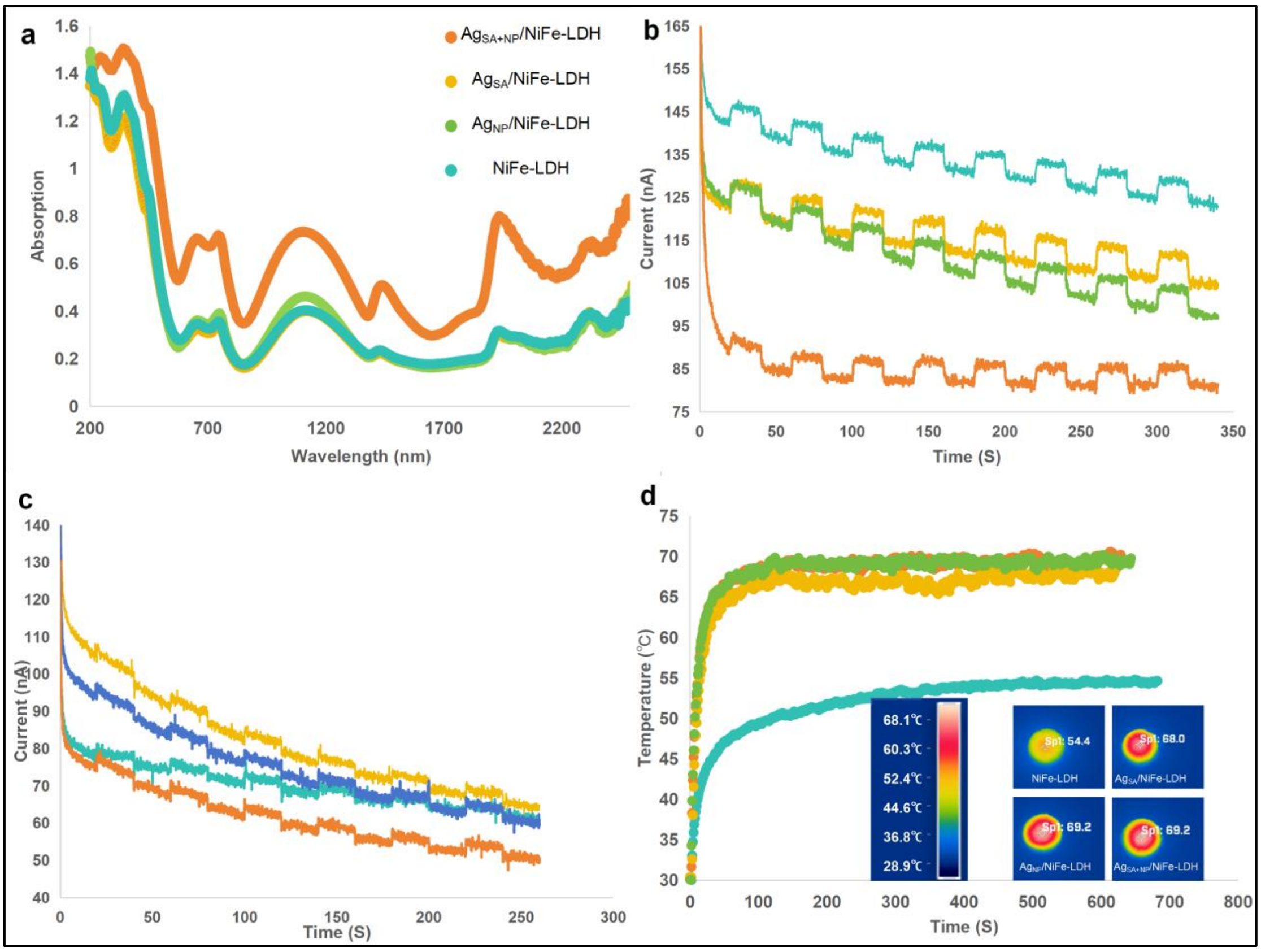


**a,** UV-Vis-NIR DRS: NiFe-LDH shows absorption peaks at ~500 nm and ~900 nm; $Ag_{SA}$/NiFe-LDH maintains spectral profiles vs. NiFe-LDH; $Ag_{NP}$/NiFe-LDH exhibits enhanced NIR absorption; $Ag_{SA+NP}$/NiFe-LDH demonstrates broadband enhancement across visible-NIR regions. **b,** Transient photocurrent (680 nm): NiFe-LDH generates baseline response; $Ag_{SA}$/NiFe-LDH shows enhanced photoelectric activity vs. $Ag_{NP}$/NiFe-LDH; $Ag_{SA+NP}$/NiFe-LDH displays further enhancement. **c,** Transient photocurrent (808 nm): NiFe-LDH exhibits weak response; $Ag_{SA}$/NiFe-LDH achieves higher photocurrent than $Ag_{NP}$/NiFe-LDH; $Ag_{SA+NP}$/NiFe-LDH maintains enhanced performance. **d,** Photothermal conversion: NiFe-LDH displays limited efficiency; $Ag_{NP}$/NiFe-LDH outperforms $Ag_{SA}$/NiFe-LDH; $Ag_{SA+NP}$/NiFe-LDH shows maximized

photothermal enhancement.

**Fig. S4. Stability of the photosensitive properties of $Ag_{SA+NP}$/NiFe-LDH under physiological conditions or during NIR irradiation.**

Physiological conditions were set as $Ag_{SA+NP}$/NiFe-LDH composite incubated in PBS buffer (pH 7.4) at 37°C for 24 hours, and NIR was set as the material was subjected to 10 cycles of NIR irradiation (808 nm, 1 W/cm², 5 min on /5 min off).

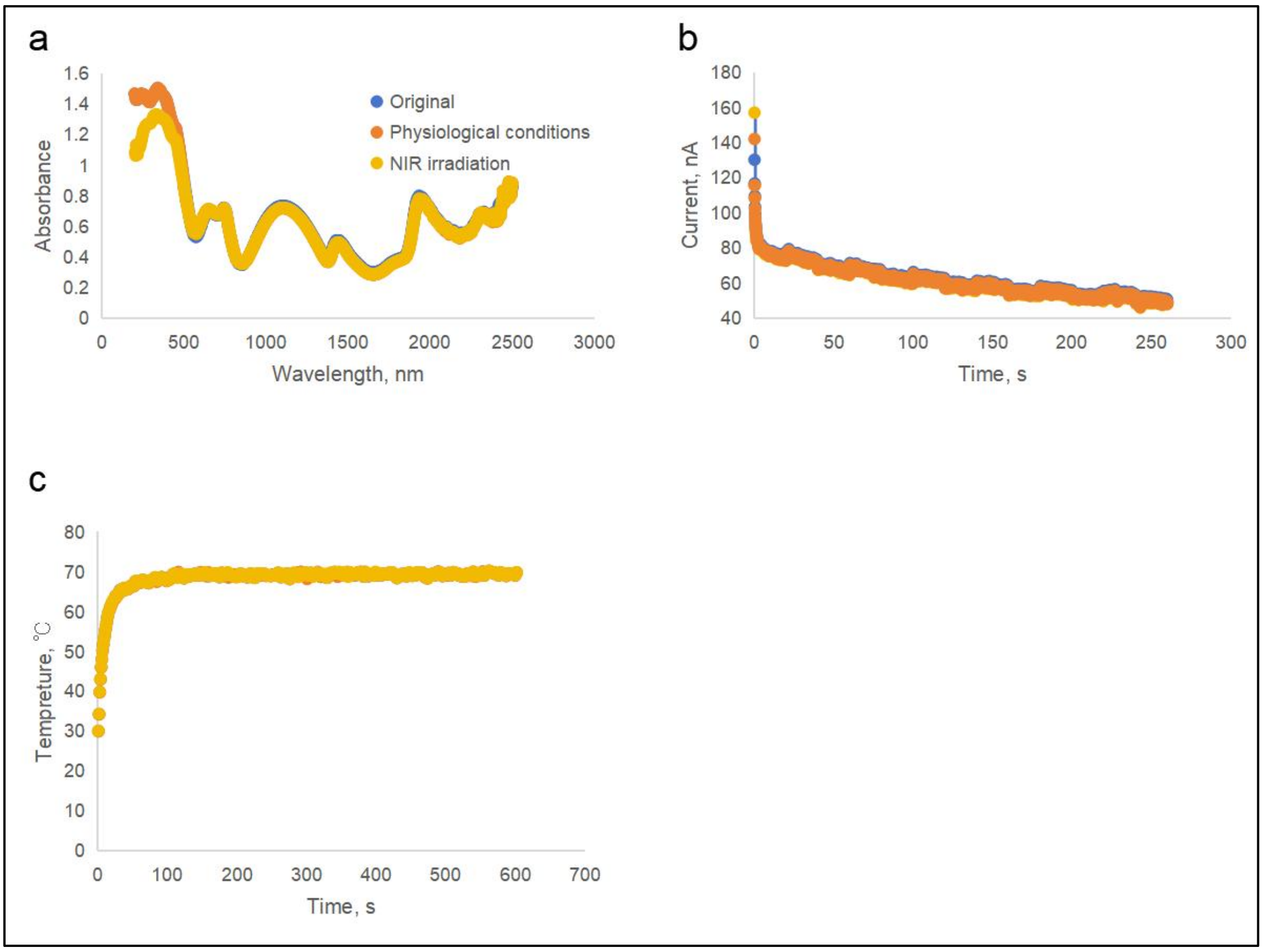


a, UV-vis-NIR absorption spectra of the material before and after physiological conditions or NIR irradiation, showing no significant change. b, Transient photocurrent responses at 808 nm before and after physiological incubation or NIR cycling, demonstrating retained photoelectric activity. c, Photothermal heating curves under 808 nm irradiation before and after treatments, showing consistent photothermal conversion. These results confirm the robustness of the Ag SA+NP synergy under biologically relevant conditions.

**Fig. S5. Fuorescence and UV spectral analyses about $Ag_{SA+NP}$/NiFe-LDH and PAs.**

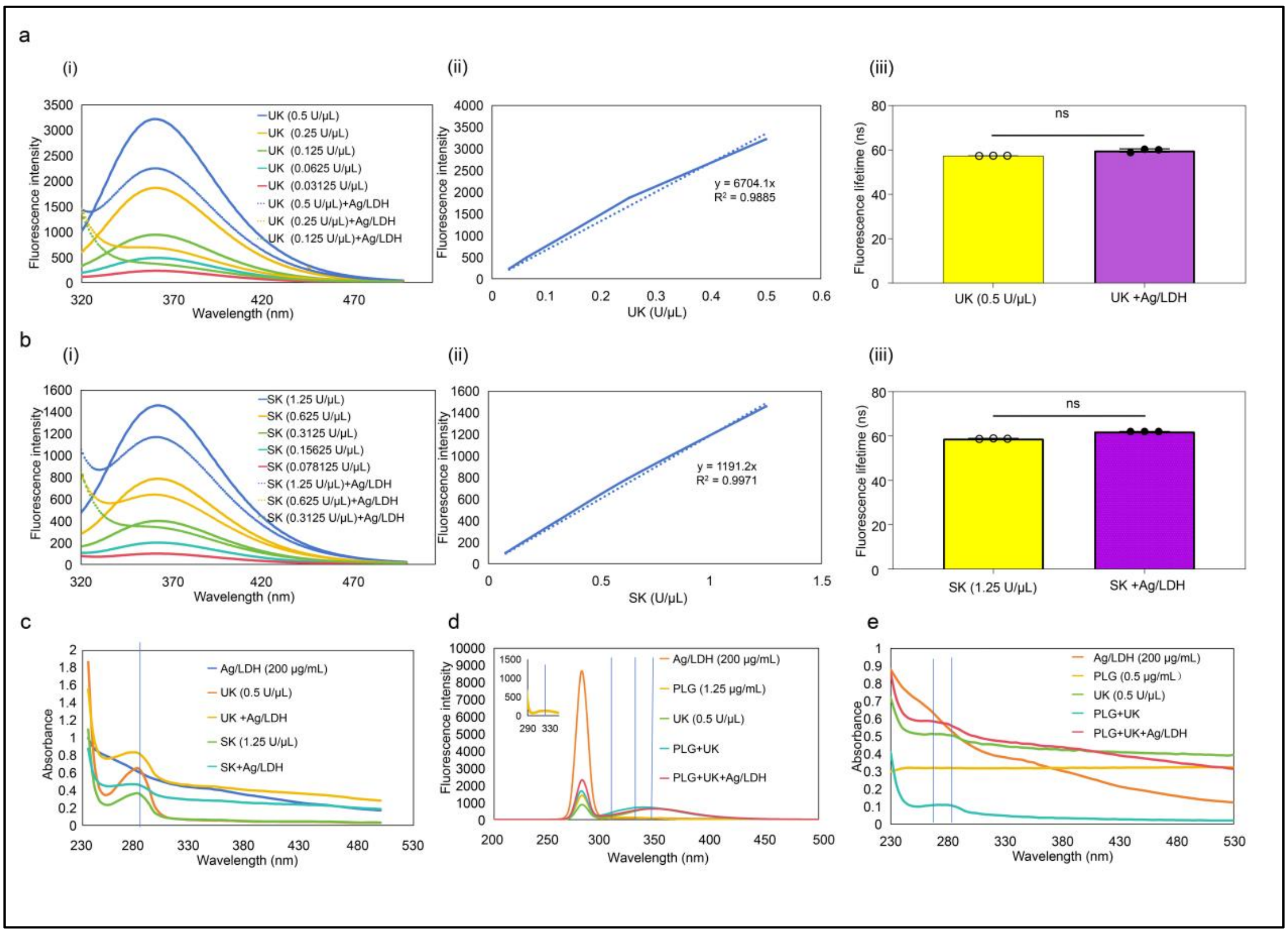


**a,** Effect of $Ag_{SA+NP}$/NiFe-LDH on UK according to the fluorescence spectrum. **(i)** Effect of concentrations and $Ag_{SA+NP}$/NiFe-LDH on the fluorescence intensity of UK. Fuorescence intensity increased as the concentrations increased, and $Ag_{SA+NP}$/NiFe-LDH (200μg/mL) could quench the fluorescence of UK. **(ii)** Fluorescence intensity changed with a linear trend along with concentrations of UK. **(iii)** $Ag_{SA+NP}$/NiFe-LDH had no significant effect on the fluorescence lifetime of UK, indicating static quenching, data are presented as mean ± SD (n = 3). **b,** Effect of $Ag_{SA+NP}$/NiFe-LDH on SK using the fluorescence spectrum. **(i)** Effect of concentrations and $Ag_{SA+NP}$/NiFe-LDH on the fluorescence intensity of SK. The fluorescence intensity increased as the concentration increased, and

$Ag_{SA+NP}$/NiFe-LDH (200μg/mL) could quench the fluorescence of SK. **(ii)** Fluorescence intensity changed with a linear trend along with SK concentrations. **(iii)** $Ag_{SA+NP}$/NiFe-LDH had no significant effect on the fluorescence lifetime of SK, indicating static quenching, data are presented as mean ± SD (n = 3). **c,** Effect of absorbance of $Ag_{SA+NP}$/NiFe-LDH on UK and SK. The peak positions of UK and SK did not change with/without $Ag_{SA+NP}$/NiFe-LDH. **d,** Effect of $Ag_{SA+NP}$/NiFe-LDH on UK and plasminogen fusion protein (PLG) evaluated by the fluorescence spectrum. The plasminogen fusion protein had an emission peak around 320 nm, while UK had an emission peak around 350 nm, the combination of UK and plasminogen fusion protein had an emission peak around 340 nm, and the combination of $Ag_{SA+NP}$/NiFe-LDH, UK, and plasminogen fusion protein had an emission peak around 350 nm, indicating $Ag_{SA+NP}$/NiFe-LDH could inhibit the combination of UK and plasminogen. **e,** Effect of $Ag_{SA+NP}$/NiFe-LDH on UK and PLG evaluated by the UV spectra. Plasminogen fusion protein had no absorption peak, UK had an absorption peak around 260 nm, the combination of UK and plasminogen fusion protein had an absorption peak around 280 nm, and the combination of $Ag_{SA+NP}$/NiFe-LDH, UK, and plasminogen fusion protein had an absorption peak around 260 nm, indicating $Ag_{SA+NP}$/NiFe-LDH could inhibit the combination of UK and plasminogen. (Fisher's exact test, $^{ns}P > 0.05$).

**Fig. S6. Whole-blood level effects of $Ag_{SA+NP}$/NiFe-LDH on plasminogen activators (PAs).**

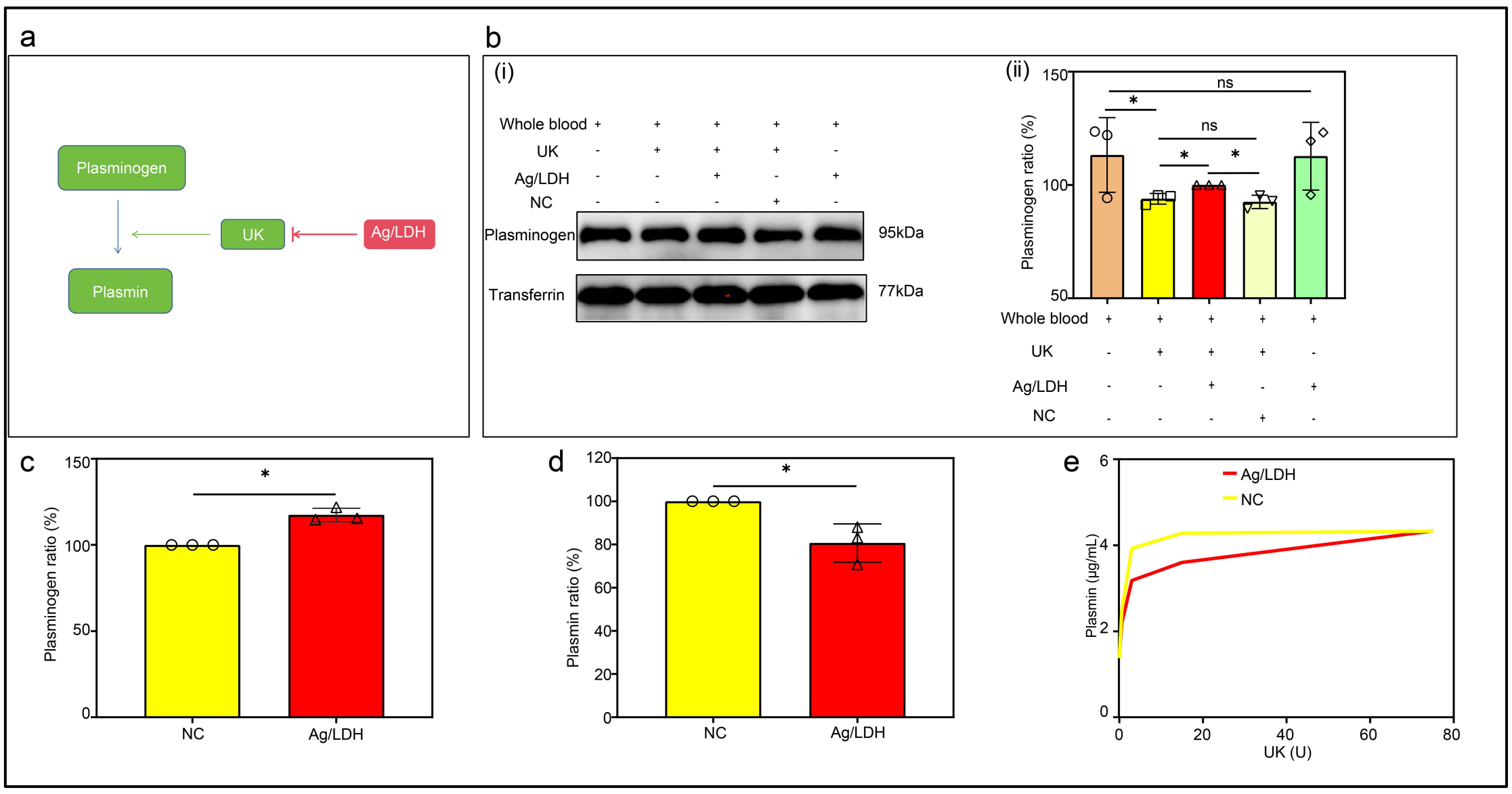


**a,** Mechanism schematic: $Ag_{SA+NP}$/NiFe-LDH inhibits UK-mediated plasminogen activation to plasmin; **b,** WB analysis: **(i)** Band patterns: UK reduces residual plasminogen vs. whole blood (Lane 1→2); $Ag_{SA+NP}$/NiFe-LDH attenuates this reduction (Lane 3) vs. NC (Lane 4); Minimal effect on whole blood alone (Lane 5), indicating that the residual concentrations of plasminogen in $Ag_{SA+NP}$/NiFe-LDH solution sample were higher than the NC sample; **(ii)** Densitometry confirms band trends; **c,** ELISA: Higher residual plasminogen with $Ag_{SA+NP}$/NiFe-LDH vs. NC; **d,** Chromogenic assay: Lower plasmin concentration with $Ag_{SA+NP}$/NiFe-LDH vs. NC; **e,** ELISA: $Ag_{SA+NP}$/NiFe-LDH reduces plasmin generation at low UK concentrations, with diminishing effects at high UK concentrations (fixed whole blood/material volume). The part of work testified that $Ag_{SA+NP}$/NiFe-LDH reduces the transformation from plasminogen to plasmin, data are presented as mean ± SD (n = 3), (Fisher's exact test. $^{ns}P > 0.05$, $^{*}P < 0.05$ ).

**Fig. S7. Effect of $Ag_{SA+NP}$/NiFe-LDH on coagulation parameters**

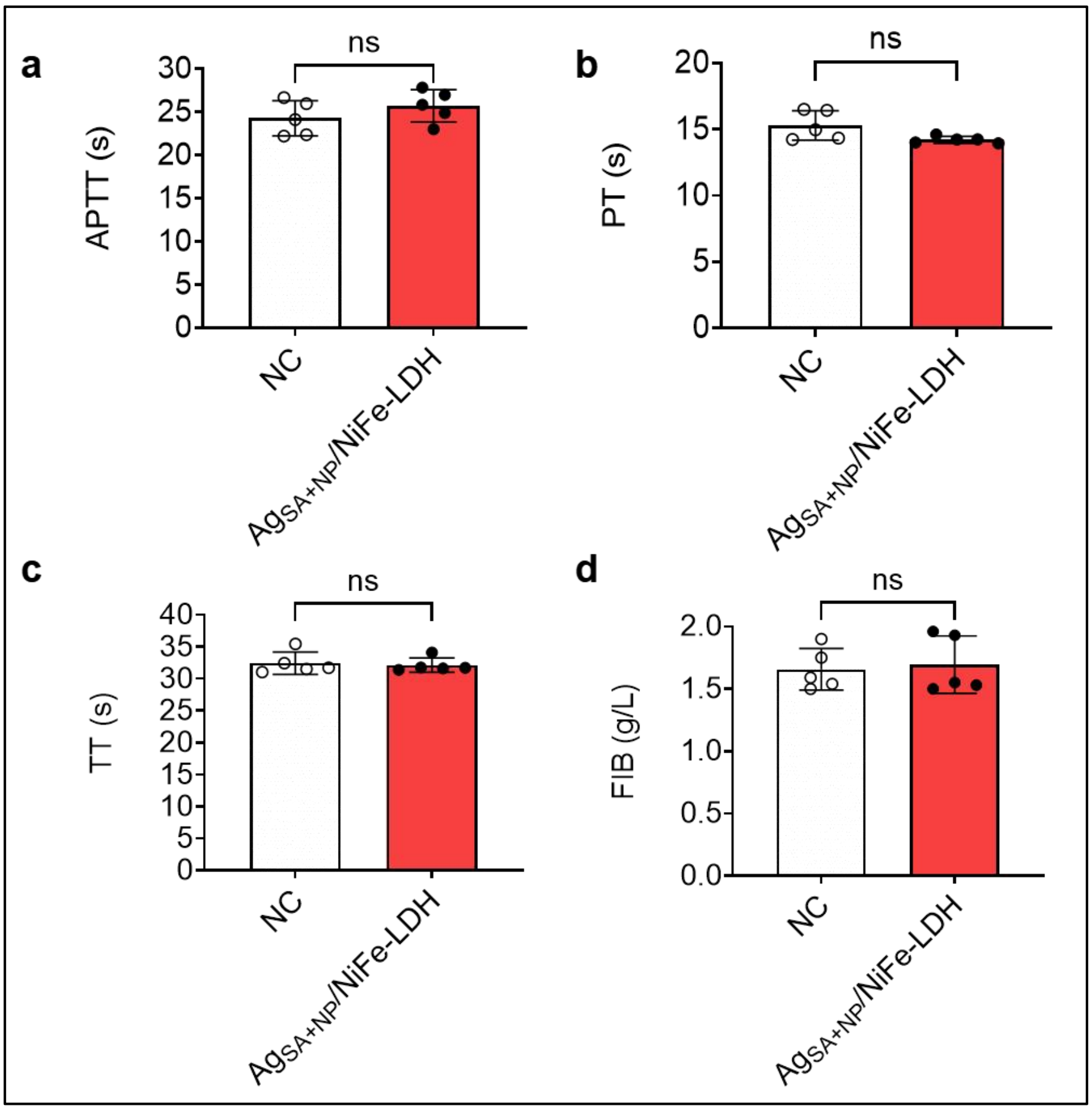


a, activated partial thromboplastin time (APTT), b, prothrombin time (PT), c, thrombin time (TT), and fibrinogen (FIB), data are presented as mean ± SD (n = 5), were measured in rat plasma after incubation with 800 μg/mL $Ag_{SA+NP}$/NiFe-LDH or negative control (NC). No significant differences were observed ($^{ns}P > 0.05$, Fisher's exact test), indicating that the material does not interfere with the coagulation cascade.

**Fig. S8**. **Acute toxicity assessment of $Ag_{SA+NP}$/NiFe-LDH.**

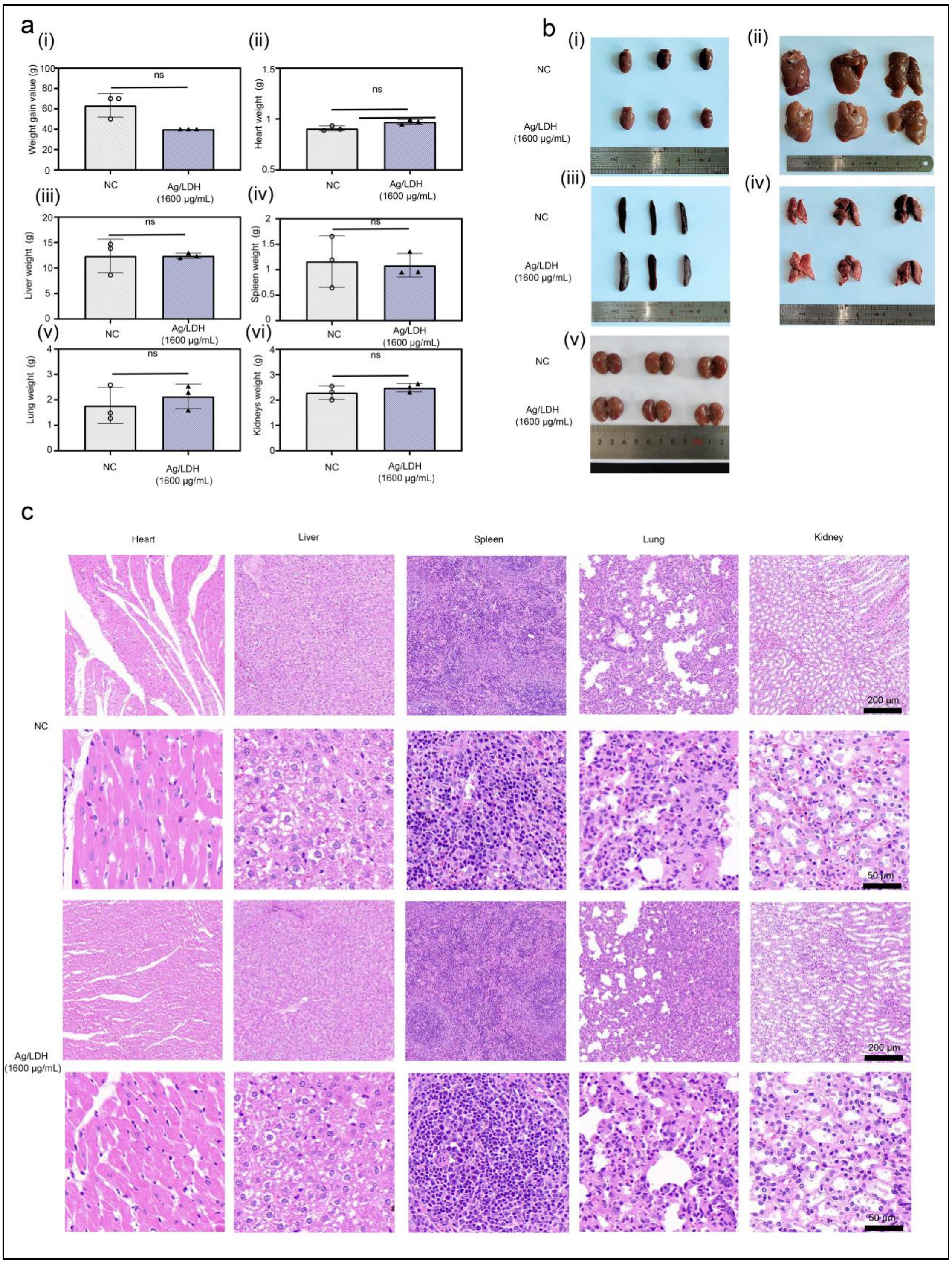


**a,** No significant differences vs. NC in: body weight gain (i), heart (ii), liver (iii), spleen (iv), lung (v), or kidney weights (vi); **b,** Representative organ photos: heart(i), liver(ii), spleen(iii), lung(iv), kidneys(v); **c,** H&E histology in representative organs.

The part of work testified the safety of $Ag_{SA+NP}$/NiFe-LDH when intravenous injection**,** data are presented as mean ± SD (n = 3), (Fisher’s exact test, $^{ns}P$>0.05)

**Fig. S9. Hematological and serum biochemical analysis after 14 days after intravenous administration of $Ag_{SA+NP}$/NiFe-LDH (1600 μg/mL, 1.43 μL/g) or NC in rats.**

**Fig. S9-1. Hematological analysis.**

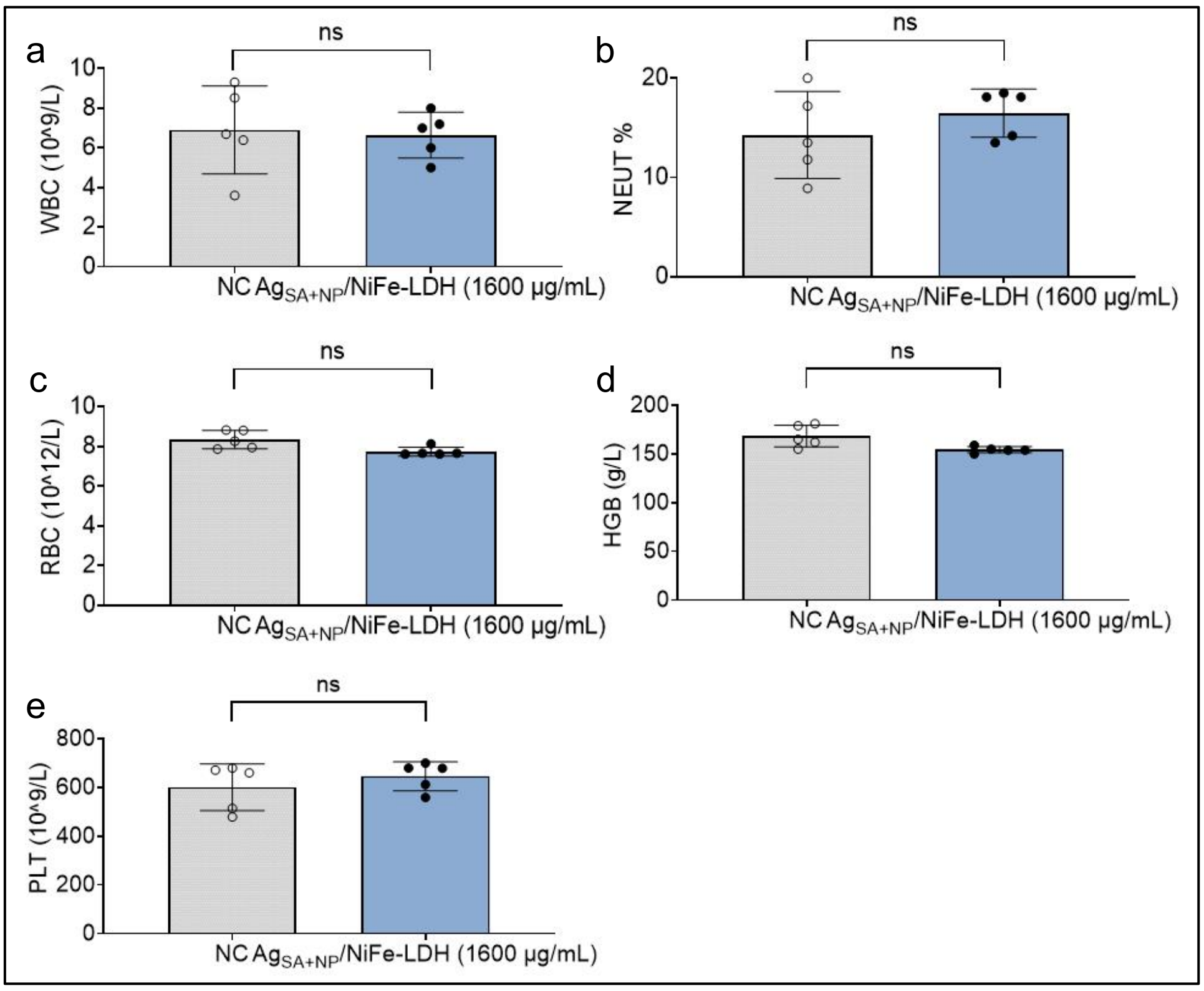


Compared with the NC group, there was no significant difference in white blood cell (a), neutrophil percentage (b), red blood cell (c), hemoglobin (d), and platelet (e) between the $Ag_{SA+NP}$/NiFe-LDH group and the NC group, data are presented as mean ± SD (n = 5), (two-way ANOVA, $^{ns}P > 0.05$).

**Fig. S9-2. Serum biochemical analysis.**

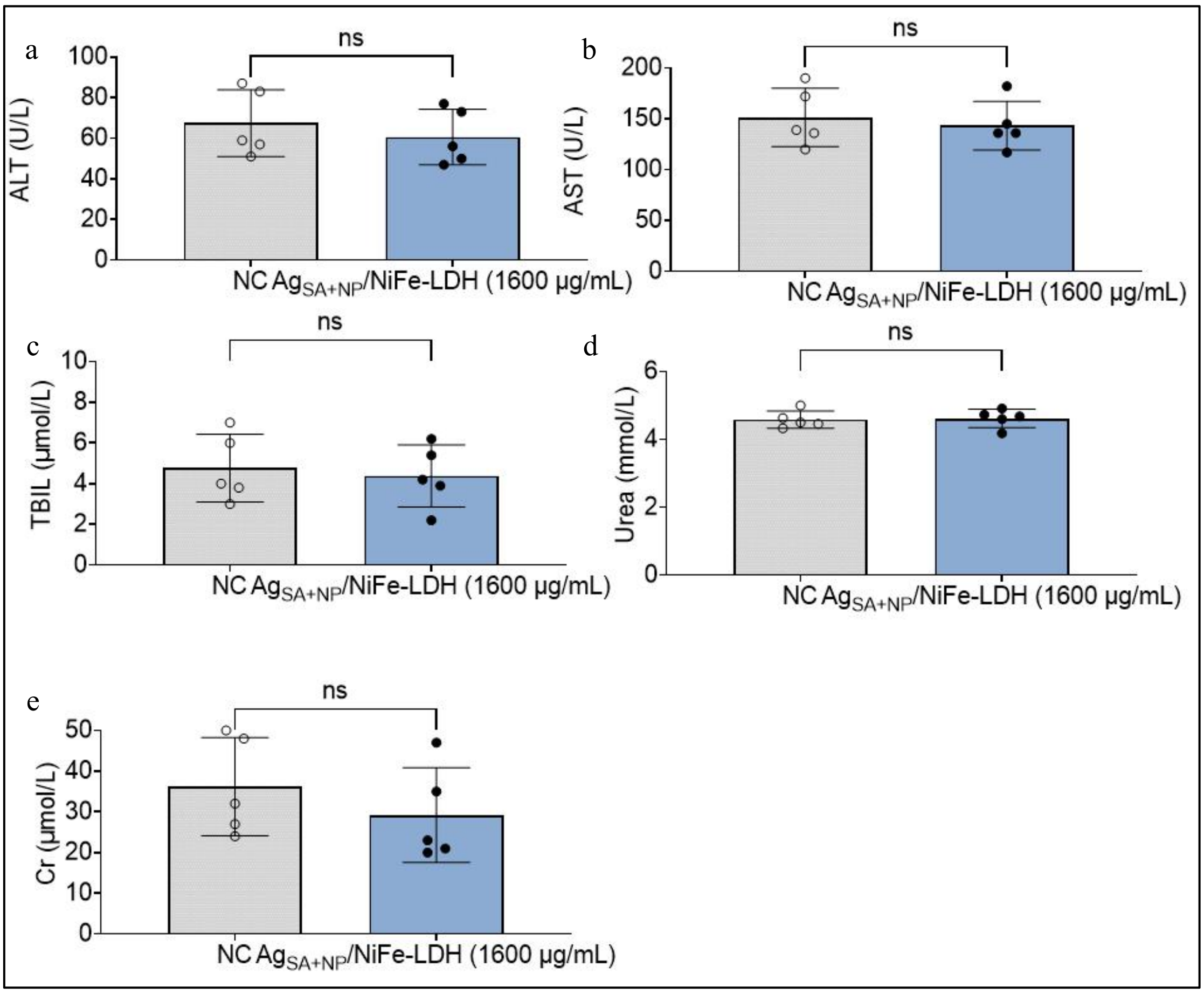


Compared with the NC group, there were no significant differences in alanine aminotransferase (a), aspartate aminotransferase (b), total bilirubin (c), urea (d), and creatinine (e) in the Ag$_{SA+NP}$/NiFe-LDH group, data are presented as mean ± SD (n = 5), (two-way ANOVA, $^{ns}P > 0.05$).

**Fig. S10. In vivo biodistribution and clearance of $Ag_{SA+NP}$/NiFe-LDH.**

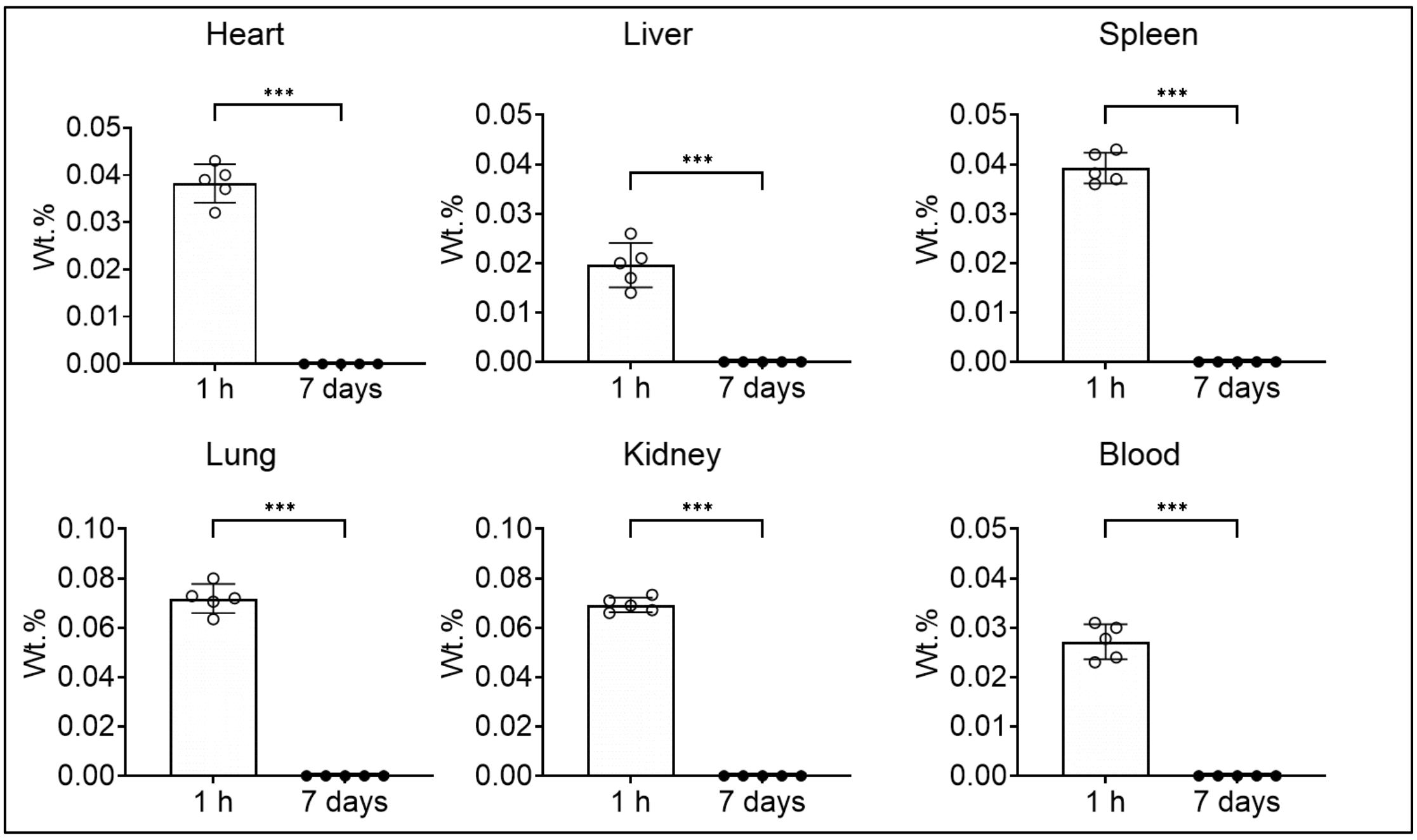


Ni content in major organs (heart, liver, spleen, lungs, kidneys) and blood was measured by ICP at indicated time points after intravenous injection (1600 μg/mL, 1.43 μL/g) in rats. Relative Ni content in whole body at 1 h, and 7 days post-injection, demonstrating >99% clearance by 1 week, data are presented as mean ± SD (n = 5), (Fisher's exact test, $^{***}P$<0.001).

To assess the fate of the NiFe-LDH-based material after therapeutic action, we investigated its biodistribution and clearance in mice over 7 days. Ni content was measured by ICP as a proxy for the material, since Ag levels were below reliable detection limits and Fe has a high endogenous background. As shown in Figure S14a, at 1 h post-injection, the material distributed primarily to the lungs and kidneys, with lower accumulation in the liver and spleen. This distribution pattern differs from typical nanoparticle sequestration by the reticuloendothelial system and suggests that the material may bypass or rapidly transit through the liver.

By day 7, Ni levels in all major organs and whole blood had decreased dramatically to approximately 1% of the 1 h value, indicating rapid and efficient clearance from the body. The highest residual signals were still observed in the lungs and kidneys, implying that these organs may serve as elimination routes—possibly via renal excretion into urine or through pulmonary clearance mechanisms. Given that over 99% of the accumulated material was eliminated within one week, with residual levels approaching the ICP detection limit, longer-term tracking was not pursued, as the remaining amounts pose negligible risk of chronic retention.

These results demonstrate that the material exhibits minimal long-term accumulation and is rapidly cleared from the body, supporting its potential for clinical translation. Future studies will focus on identifying the exact excretion pathway (urine vs. feces) and characterizing the chemical form of the cleared material.

**Fig. S11. Dose- and time-dependent hemostatic effects of $Ag_{SA+NP}$/NiFe-LDH.**

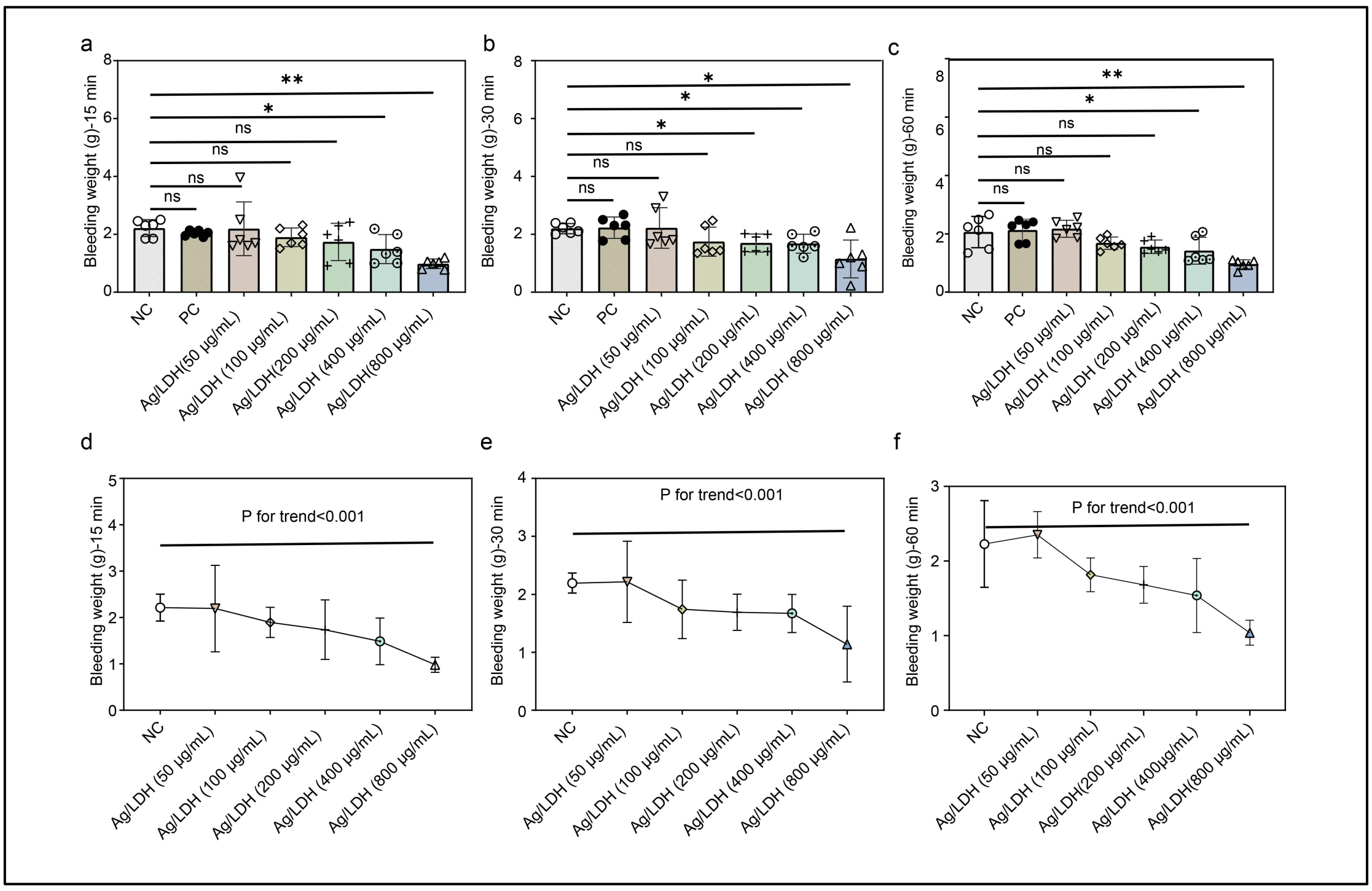


**a,** 15 min circulation: 400/800 μg/mL significantly reduce bleeding weight vs. NC; **b,** 30 min circulation: 200/400/800 μg/mL reduce bleeding weight vs. NC; **c,** 60 min circulation: 400/800 μg/mL reduce bleeding weight vs. NC; **d,** 15 min: dose-dependent decrease with increasing concentrations; **e,** 30 min: dose-dependent decrease with increasing concentrations; **f,** 60 min: dose-dependent decrease with increasing concentrations. The part of work testified that the $Ag_{SA+NP}$/NiFe-LDH has the hemostasis effect, along with concentrations, no matter the internal circulation times, data are presented as mean ± SD (n = 5). (Fisher's exact test; Chi square trend test for the trend, $^{ns}P$>0.05).

**Fig. S12. The results of differential scanning calorimeter,**

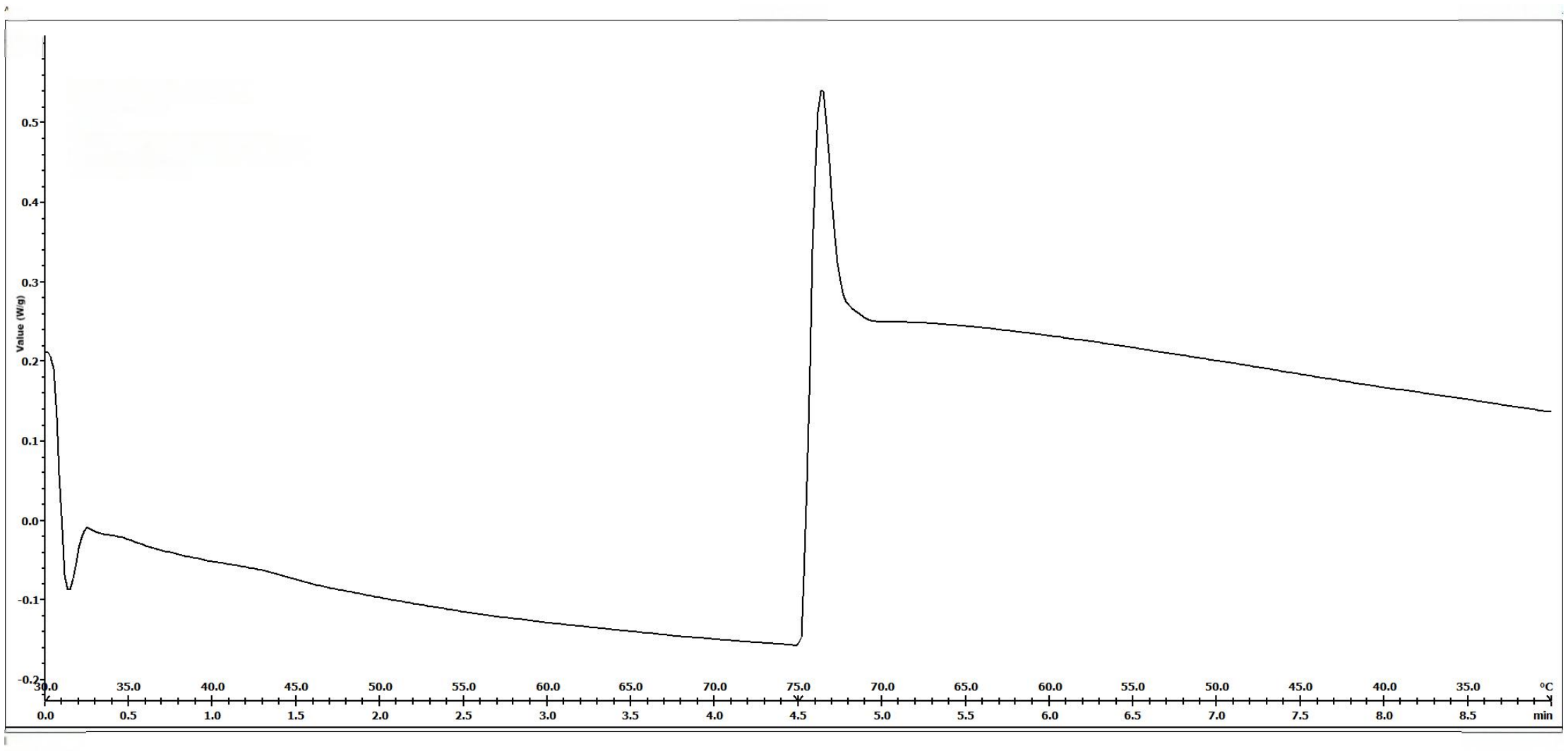


$Ag_{SA+NP}$/NiFe-LDH exhibits an increasing specific heat capacity with rising temperature, indicating that the temperature increases quickly at initial to realize instant response, and increases slowly under high temperature conditions to avoid adverse reactions.

**Fig. S13. Mechanistic validation of NIR-switchable hemostasis and thrombolysis.**

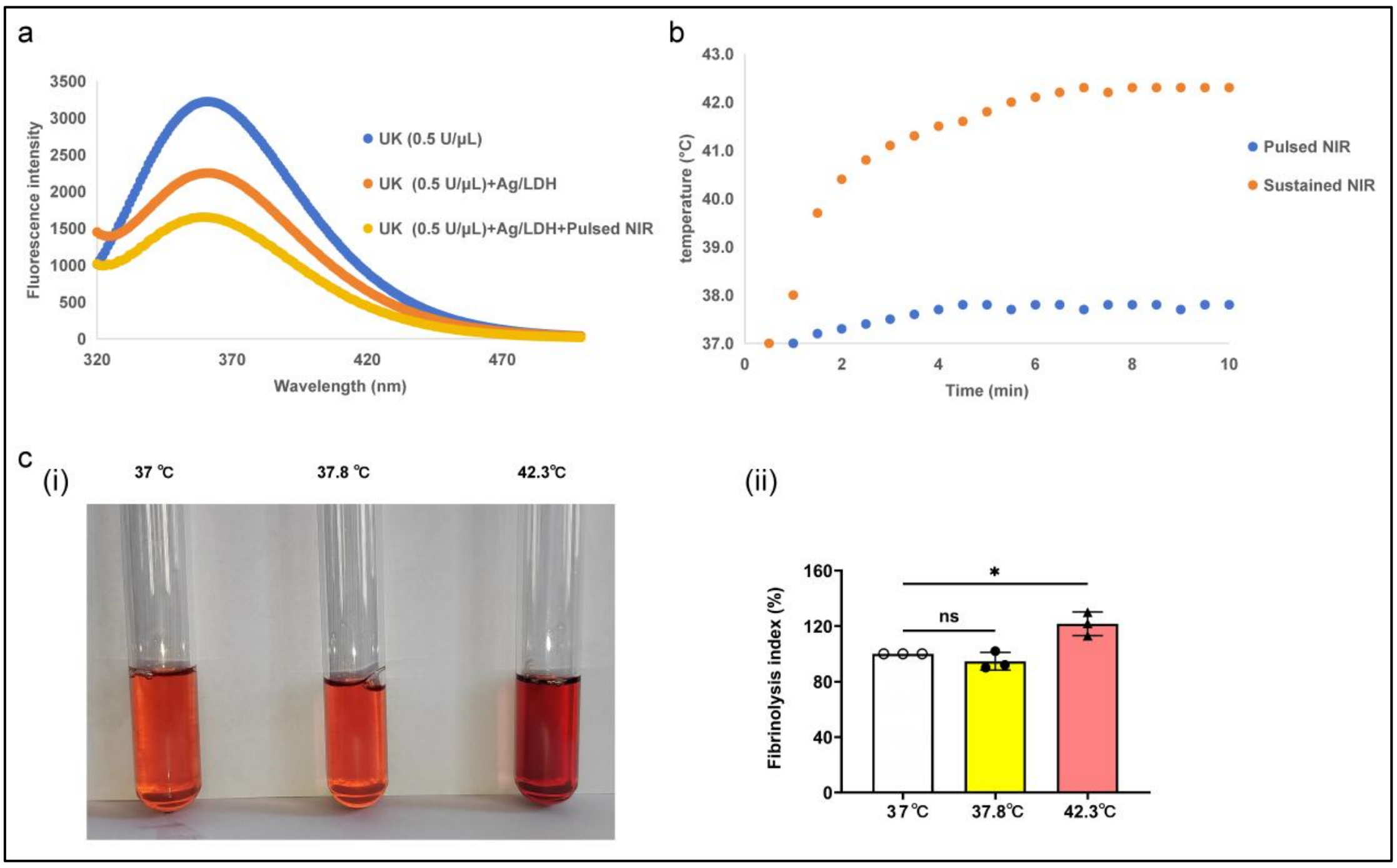


a, Fluorescence emission spectra of UK ($\lambda_{ex}$=285 nm) in the presence of $Ag_{SA+NP}$/NiFe-LDH under dark and pulsed NIR (808 nm). Sustained NIR was not tested to avoid thermal interference with the fluorescence readout; b. Temperature evolution of the fibrinolysis system under pulsed (1 s on/5 s off) and sustained 808 nm irradiation (0.1 W cm$^{-2}$, 10 min); c. Fibrinolysis assay performed without $Ag_{SA+NP}$/NiFe-LDH at the temperatures reached under pulsed (37.8 °C) and sustained NIR (42.3 °C), indicating that the mild temperature increase under pulsed NIR is insufficient to inhibit fibrinolysis. (i) Representative photographs of post-fibrinolysis supernatants. (ii) Fibrinolysis index, data are presented as mean ± SD (n = 3). (one-sample t-test against 100%), $^{ns}P$>0.05, and $^{*}P$<0.05.

**Fig. S14. NIR-Switchable hemostasis and thrombolysis in human blood samples.**

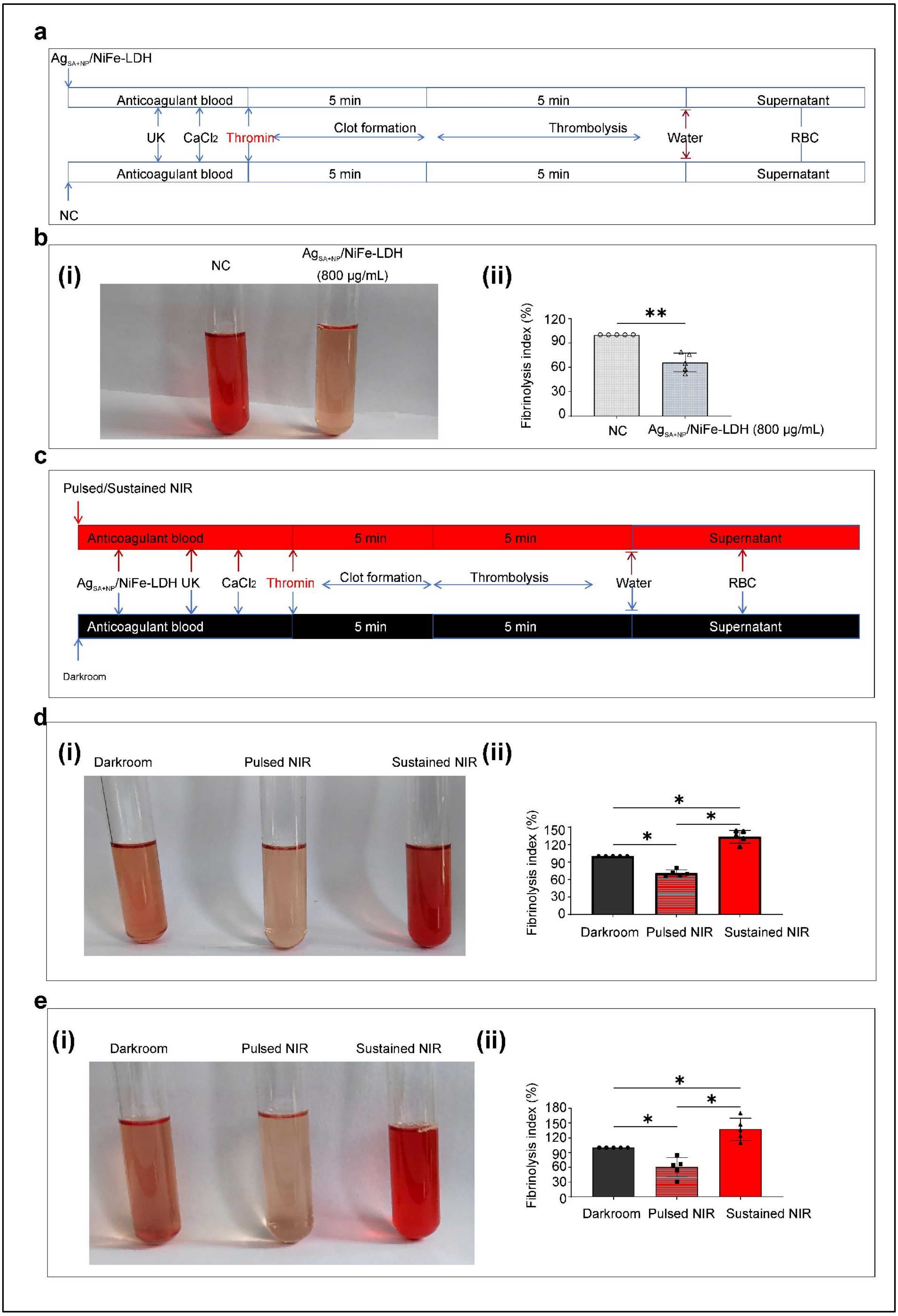

**a,** Schematic of fibrinolysis assays for $Ag_{SA+NP}$/NiFe-LDH hemostasis (healthy volunteers). **b,** Anti-fibrinolytic effect of $Ag_{SA+NP}$/NiFe-LDH. **(i)** Post-fibrinolysis supernatants: lighter color with $Ag_{SA+NP}$/NiFe-LDH vs. NC, Representative photographs; quantification is shown as fibrinolysis index in (ii). **(ii)** Fibrinolysis index: lower in $Ag_{SA+NP}$/NiFe-LDH group vs. NC. **c,** Schematic of NIR-mediated hemostasis assays. **d,** Effect of NIR illumination mode on anti-fibrinolysis in healthy volunteers. **(i)** Supernatants: lighter color in pulsed NIR group, deeper color in sustained NIR group vs. darkroom group, Representative photographs; quantification is shown as fibrinolysis index in (ii). **(ii)** Fibrinolysis index: lower with pulsed NIR, higher with sustained NIR vs. darkroom. **e,** Effect of NIR illumination mode on anti-fibrinolysis in ICH patients. **(i)** Supernatants: lighter color (pulsed NIR), deeper color (sustained NIR) vs. Darkroom, Representative photographs; quantification is shown as fibrinolysis index in (ii). **(ii)** Fibrinolysis index: lower (pulsed NIR), higher (sustained NIR) vs. Darkroom, data are presented as mean ± SD (n = 5), Fisher's exact test were used to test group differences. $^{*}P < 0.05$, $^{**}P < 0.01$.
